# Phenotypic Profiling of Anchote (*Coccinia abyssinica* (Lam.) Cogn.) Accessions Through Agro-Morphological and Physiological Markers

**DOI:** 10.3390/plants14152334

**Published:** 2025-07-28

**Authors:** Dejene Bekele Dibaba, Temesgen Magule Olango, Bizuayehu Tesfaye Asfaw, Desta Fikadu Mijena, Meseret Tesema Terfa

**Affiliations:** 1 Ethiopian Institute of Agricultural Research, Wondo Genet Agricultural Research Center, Shashemene P.O. Box 198, Ethiopia; dejukominew@gmail.com; 2 School of Plant and Horticultural Science, Hawassa University, Hawassa P.O. Box 05, Ethiopia; temeolait@gmail.com (T.M.O.); tbizuayehu@gmail.com (B.T.A.); 3 Ethiopian Institute of Agricultural Research, Debre Zeit Agricultural Research Center, Debre Zeit P.O. Box 32, Ethiopia; desdar2008@gmail.com

**Keywords:** phenotyping, agro-morphological, physiological, qualitative, quantitative, accessions, anchote (*Coccinia abyssinica* (Lam.) Cogn.)

## Abstract

Anchote (*Coccinia abyssinica*) is a neglected high-potential food and nutrition security tuber crop in Ethiopia. Phenotyping core germplasm collections using agro-morphological and physiological markers is essential for effective crop improvement and utilization. A total of 282 anchote germplasms were profiled using six qualitative and twenty-six quantitative agro-morphological and physiological traits. Augmented Block Design was used for the experiment at the Debre Zeit Agricultural Research Center. The chi-square test and Shannon diversity index indicated the presence of substantial phenotypic variation and diversity among the accessions based on the predominant qualitative traits studied. The quantitative agro-morphological and physiological traits showed wider variability and ranges for the accessions. The broad-sense heritability and genetic advance as a percentage of the mean were notably high for quantitative traits such as root yield, vine length, and leaf area index. A significantly positive correlation was observed among agronomically important traits such as root yield and root diameter as well as root yield and leaf area. The principal component analysis for qualitative and quantitative traits found that ten components explained 72.2% of the variation for qualitative traits, whereas nine components accounted for 69.96% of the variation in quantitative traits. The primary contributors to the variations are traits such as root (shape, flesh color, and yield), leaf (color, length, diameter, area) and fruit (length, diameter, and weight). Further, the accessions were grouped into two and three clusters based on qualitative and quantitative traits, respectively, indicating that quantitative characters better differentiated among the accessions. Similarly, the tanglegram showed little similarity between the qualitative and quantitative agro-morphological and physiological traits in clustering the accessions. These findings indicate the presence of sizable trait variation among the accessions that can be exploited as a selection marker to design and facilitate conservation and breeding strategies of anchote.

## 1. Introduction

Root and tuber crops make a vital contribution to nutrition and food security by providing energy-rich food in many smallholder farming systems of sub-Saharan Africa [1]. Among them, anchote (*Coccinia abyssinica*), an orphan root crop native to Ethiopia, holds significant potential for sustainable agriculture [2,3].

Anchote (*Coccinia abyssinica* (Lam.) Cogn.) is a diploid (2x = 20) species that belongs to the Cucurbitaceae family and genus Coccinia [4]. This genus comprises 27 species confined to sub-Saharan Africa [5]. It is domesticated and remains indigenously grown in large portions of South, Southwest, and Western Ethiopia. Anchote has edible tubers varying in shape that have spherical to conical shapes at maturity [4]. In different growing zones of the country, it is recognized by different vernacular names such as anchote (Afan Oromo), Ushushe (Wolayita), and Shushe/Ushushe (Dawuro) [6]. The crop grows in varying ecological niches with altitudinal ranges from 300 to 2800 m above sea level and average annual rainfall ranging from 762 to 1016 mm [7,8]. This is indicative of the presence of varying adaptability and diversity in the anchote germplasm.

Anchote is valued for its high nutritional content and adaptability to a wide range of growing environments [4,9]. It is a versatile tuber crop used for food and feed, and it holds significance in socio-cultural rituals and livelihoods in Ethiopia [3,10]. Recent studies reported that anchote is abundant in carbohydrates and dietary fiber [11], with notable levels of essential minerals such as calcium [12], Protein [13], Iron, and Phosphate [9,13]. It has industrial importance in which the starch, pectin, can be utilized in strawberry jam preparation by replacing the commercial pectin [14,15], and the flour is shown to be incorporated with wheat to develop bakery products [16]. Given its nutritional and industrial potential, interest in anchote has grown over the past two decades.

Nonetheless, anchote is cultivated predominantly by smallholder farmers on a small scale for domestic use. Compared with other major root and tuber crops such as potato, cassava, and sweet potato, large-scale production and industrial utilization remain insignificant despite being rich in micronutrients (especially calcium) [17] and suitable for medicinal use [18]. Although Ethiopia is a center of diversity and domestication for anchote, it has remained an underutilized orphan crop with limited crop conservation, maintenance, and improvement in contemporary agricultural practices due to the absence of attention from agricultural research and development, and largely, the lack of inclusive agricultural policy for indigenous food crops [19]. In addition to the little effort from research and development, one of the important challenges for anchote’s improvement is the lack of understanding and characterization of its genetic diversity. A thorough characterization of germplasm stocks is a crucial initial phase for improvement programs, including for anchote [20]. Genetic diversity is an important element for population survival, evolutionary dynamics, genetic enhancement, and adaptation to shifting climatic conditions [21]. Understanding and exploring genetic diversity lays a roadmap and framework for designing breeding programs [22]. The information obtained from these characterizations helps breeders to exploit various plant characteristics, ultimately contributing to the enhancement of crop features and properties [23] and strategic conservation and maintenance of the germplasms with unique characters [24]. This has paramount significance, especially for underutilized and orphan crops such as anchote.

Morphological markers are powerful, easy, and relatively cheap markers to profile and screen genotypes from populations and to help document variation in the phenotypic traits [22,25]. They are heritable characters that can be assessed both visually and metrically using qualitative and quantitative scores. Examples of such traits include leaf shape, color, or size; flower color or number; seed color or size; plant stature; and below-ground features like root number, size, shape, or color [26]. These morphological markers serve as a valuable tool in genetic mapping and selection in plant breeding, facilitating rapid germplasm evaluation and characterization. The benefits of using these markers include the absence of a need for specialized equipment and their direct relevance to agronomy. However, their usefulness across the genome is limited by factors such as environmental sensitivity, pleiotropy, and a restricted number of observable traits [26,27].

Several past research results using different quantitative and qualitative morphological markers indicated the existence of variations in anchote accessions [3,9,19,28]. Despite notable efforts in the characterization of anchote using these agro-morphological traits, there remain discrepancies in the number of accessions included, the type, and the number of traits studied. In this study, we used the classical quantitative and qualitative agro-morphological traits and additional physiological stress indicators to assess the genetic diversity and the trait associations.

## 2. Results

### 2.1. Agro-Morphological Trait Diversity of Anchote Accessions Based on Qualitative Traits

**Figure 1:**
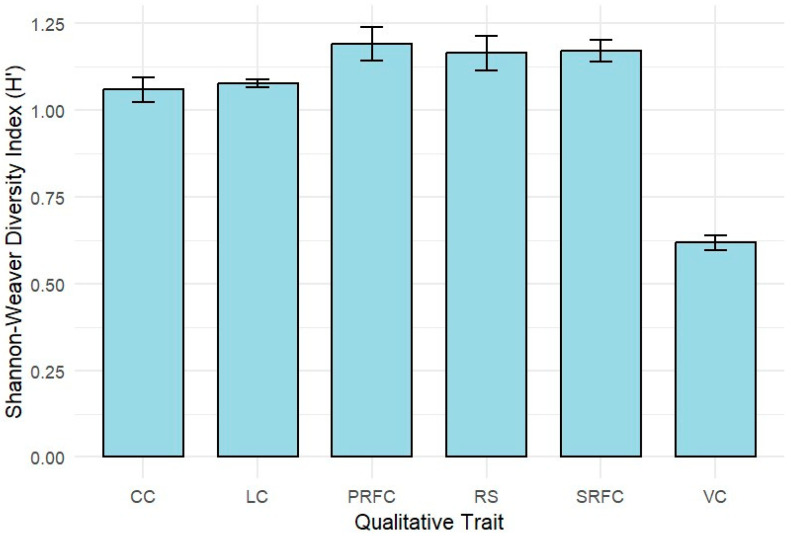
Shannon–Weaver diversity index for six qualitative morphological traits for the 282 accessions of anchote: root shape (RS), predominant root flesh color (PRFC), secondary root flesh color (SRFC), vine color (VC), leaf color (LC), and canopy coverage (CC).

The qualitative trait diversity for the 282 anchote accessions was studied based on the Shannon–Weaver diversity indices (H′) for six morphological trait classes and their subclasses (Figure 1; Table 1). Predominant root flesh color (1.23) exhibited the highest Shannon–Weaver diversity index, followed by root shape (1.18), secondary root flesh color (1.17), leaf color (1.08), and ground coverage (1.06); in contrast, the lowest diversity index was 0.63 for the vine color.

**Figure 2:**
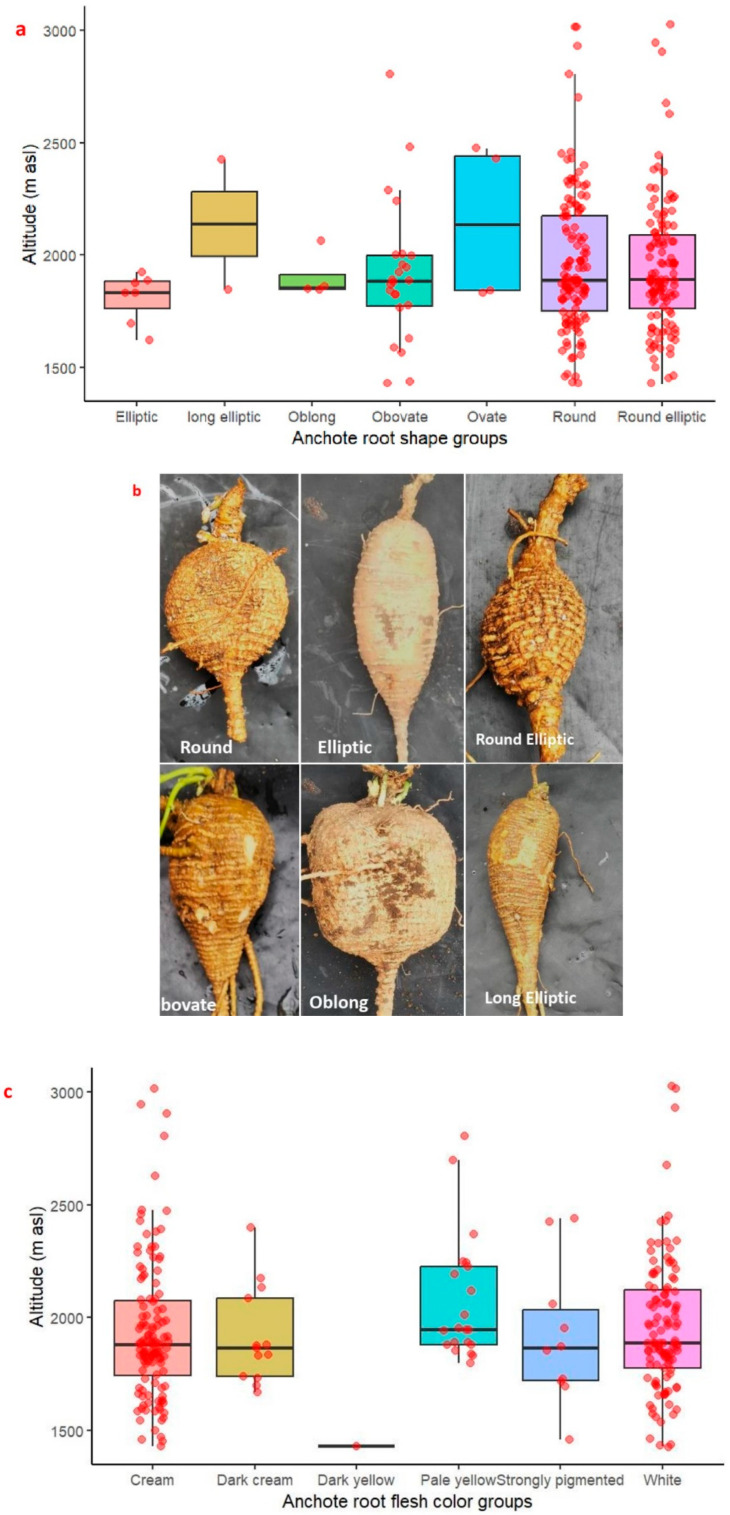

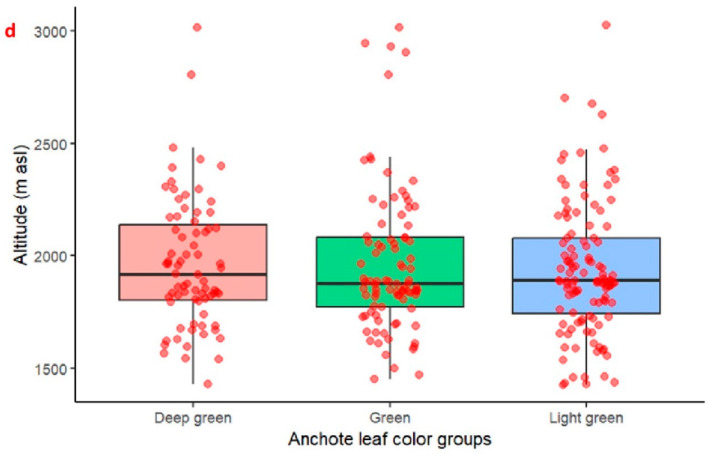
Economically important qualitative traits of 282 anchote accessions collected from different altitude levels: (**a**,**b**) root shape, (**c**) root flesh color, and (**d**) leaf color. The dots indicate the distribution of the accessions.

The results indicated that the principal root shape subclass is round shape, encompassing 126 accessions in the category followed by round elliptic with 115 accessions, and the remaining root shape subclasses contribute less to the diversity (Table 1). The predominant root flesh colors were creamy (43.97%) and white (40.07%), while pale yellow (7.44%), dark cream (4.60%), strongly pigmented (3.54%), and dark yellow color (0.35%) were observed less frequently in the population. Four types of secondary root flesh color were observed in this study, of which 123 accessions had cream color, 97 accessions had dark cream color, 50 accessions had pale yellow color, and the remaining 12 accessions had dark cream secondary root flesh color. The primary vine color was light green, observed in 190 accessions, and the other remaining 92 accessions had green vine color. The results indicated that the leaf color of the evaluated anchote accessions was mainly green, i.e., deep green, green, and light green, which contributed 26.59%, 32.27%, and 41.13%, respectively. The evaluated canopy coverage of the different accessions revealed two-thirds of the population had very high to high canopy cover. Furthermore, the chi-square results exhibited high phenotypic variation among the qualitative traits studied. The result showed a high phenotypic class for root shape, which is followed by predominant root flesh color, canopy coverage, secondary root flesh color, vine color, and leaf color. Most of these traits are also distributed across all altitudes (Figure 2).

### 2.2. Quantitative Agro-Morphological and Physiological Trait Variability of Anchote Accessions

The studied twenty-six quantitative agro-morphological and physiological traits exhibited wider variability for the tested anchote accessions (Table 2). Accordingly, the following ranges of variability were observed for traits such as petiole length (1.0–7.60 cm), leaf length (1.0–10.67 cm), leaf diameter (1.1–11.0 cm), internode length (5.5–15.2 cm), vine length (1.0–3.9 m), fruit length (3.2–8.60 cm), fruit diameter (2.8–5.8 cm), number of seeds per locule (10.4–29.4), number of seeds per fruit (50 to 166.60), fruit weight (164.41–678.2 gm), thousand seed weight (21.1–71.2 gm), seed yield (99.8–973.8 gm), root number per plot (1–5), root length (7.32–17.5 cm), root diameter (2.5–16.44 cm), root weight per plot (0.17 to 7 kg), 5.57 t to 233.33 t for root yield, 0.53–81.6 cm^2^ for leaf area, 0.91–2.9 for leaf area index, 13.2–59.9 for canopy density, 0.1–2.4 for gap fraction leaf area index, 24.30–79.7 for chlorophyl content, and 27–87% for normalized difference vegetative index (Table 2). Among the evaluated accessions, accession 129 exhibited the highest root weight per plot and root yield, while accession 68 recorded the lowest. Accession 348 had the highest fruit weight, whereas accession 51 showed the lowest. The highest seed yield was obtained from accession 412, in contrast to the lowest yield from accession 160. Leaf area index peaked in accession 262 and was lowest in accession 6. Regarding chlorophyll content, accession 57 had the highest value, while accession 291 recorded the lowest.

**Figure 3:**
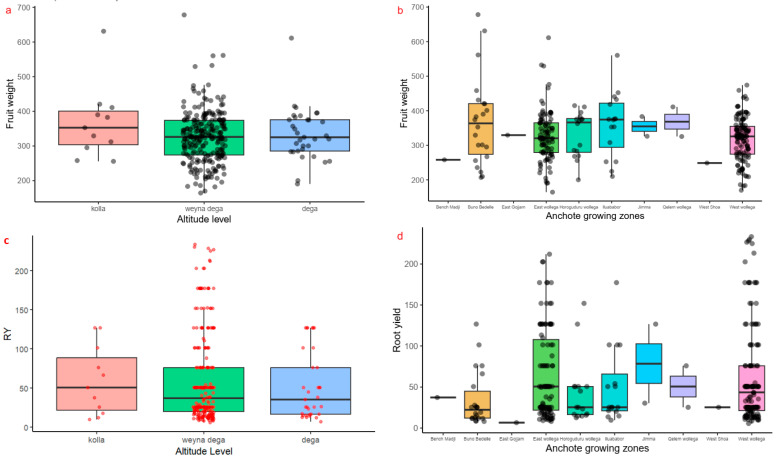

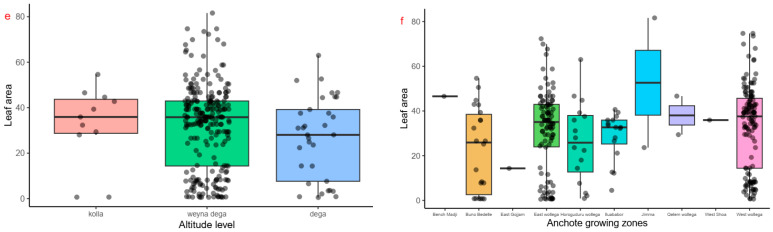
Variation in some quantitative traits such as (**a**,**b**) fruit weight, (**c**,**d**) root yield, and (**e**,**f**) leaf area of 282 anchote accessions across different altitudes (**a**,**c**,**e**), and geographic growing zones (**b**,**d**,**f**). The dots indicate the distribution of the accessions.

Furthermore, the observed coefficients of variation for the measured traits varied from 9.84% for fruit diameter to 50.8% for root weight per plot and root yield. The response of accessions for quantitative traits was not significantly affected by differences in altitude level and collection zones (Figure 3).

Genotypic coefficient of variation (GCV), phenotypic coefficient of variation (PCV), heritability in a broad sense (Hb^2^), genetic advance (GA), and genetic advance as a percentage of the mean (GAM) for 26 quantitative agro-morphological and physiological traits were evaluated. In general, estimates of PCV were slightly greater than the corresponding GCV (Table 3). High PCV and GCV values were estimated for most of the traits, particularly for RWPP, RY, and LAI. The range of PCV values was 11.49 to 92.76% for FD and RY, respectively. Similarly, the magnitude of variation in GCV for these traits ranged from 5.8 to 77.65%, respectively. The GCV and PCV values were categorized as low (0–10%), moderate (10–20%), and high (>20%) [29]. The highest PCV and GCV were observed for LL, LD, VL, SY, RNPP, RL/RD, RWPP, RY, LA, LAI, and Gap fraction leaf area index (GFLAI). On the other hand, relatively lower variances were observed for traits such as FL, FL/FD, NSPF, INL, FD, and number of seeds per locule (NSPL). On the other hand, moderate values of PCV and GCV were observed for the traits such as FL, FL/FD, NSPF, and CC.

The magnitude of broad-sense heritability values ranged between 13.85% for NSPL and 95.05% for GFLAI. It is categorized as low (0–30%), moderate (30–60%), and high (>60%) according to [30]. Accordingly, high heritability values were found for traits such as VL, SY, RY, LAI, GFLAI, and NDVI but moderate for LL, LD, INL, FL, INL/PL, FL/FD, NSPF, FW, TSW, RD, RL/RD, CD, CC, and LA. Furthermore, SY (g) and RY (t) recorded the highest value of genetic advance, 287.13% and 80.13%, respectively, indicating the existence of additive gene effect. Low GA was found for FD, NSPL, GFLAI, and INL (Table 3). A high genetic advance as the percent of the mean was recorded for RY, RWPP, SY, LAI, GFLAI, and VL in addition to their high heritability values.

### 2.3. Inter-Trait Association of Quantitative Traits

**Figure 4:**
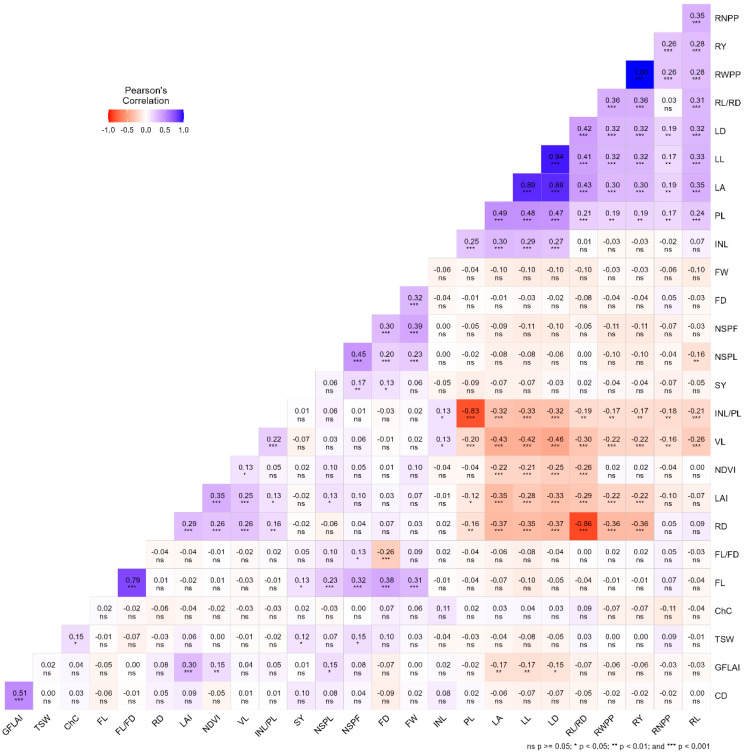
Phenotypic correlation coefficient of quantitative and physiological traits of 282 anchote accessions. PL = petiole length, LL = leaf length, LD= leaf diameter, INL = internode length, VL = vine length, INL/VL = internode length to vine length ratio, FL = fruit length, FD = fruit diameter, FL/FD = fruit length-to-diameter ratio, NSPL = number of seeds per locus, NSPF = number of seeds per fruit, FW = fruit weight, TSW = thousand seed weight, SY = seed yield, RNPP = root number per plot, RL = root length, RD = root diameter, RL/RD = root length-to-diameter ratio, RWPP = root weight per plot, RY = root yield, LA= leaf area, LAI = leaf area index, CD = canopy density, GFLAI = gap fraction leaf area index, CC = chlorophyll content, NDVI = normalized difference vegetative index.

The correlation coefficient analysis showed the existence of significant associations among some of the quantitative agro-morphological characters of anchote accessions (Figure 4). Fruit weight had a significant positive correlation with fruit length, fruit diameter, and number of seeds per fruit. Similarly, root length had a significant positive correlation with petiole length, leaf area, leaf length, leaf diameter, root length-to-diameter ratio, root yield, and root number per plot and a significant negative correlation with vine length and internode length to petiole length ratio. Root yield was associated significantly and positively with leaf area, petiole length, leaf length, leaf diameter, root length-to-diameter ratio, and root weight per plot and a significant negative correlation with vine length, root diameter, and leaf area index. Leaf area had a significant positive correlation with internode length, root weight per plot, root yield, and petiole length and a significant negative correlation with normalized difference vegetative index, vine length, and root diameter. The normalized difference vegetative index had a significant positive correlation with leaf area index and gap fraction leaf area index.

#### 2.3.1. Principal Component Analysis of the Qualitative Morphological Traits

The result from principal component analysis identified that the first nine principal components significantly contributed 72.2% of the total variation in qualitative morphological traits. PC1 was characterized as having the highest variance (14.22%) followed by PC2, PC3, PC4, and PC5, which accounted for 9.92%, 8.56%, 7.68%, 7.16%, 5.73%, 5.25%, 4.92%, 4.59%, and 4.17% of the total qualitative morphological variation, respectively. The eigenvalues, contribution rate to variability, and cumulative contribution rate are shown in Table 4.

Obovate and round elliptic root shape (RS); green and light green vine color (VC); deep green, green, and light green leaf color (LC); and high, low, and very high canopy coverage (CC) were the main contributors for total variation in PC1 (14.22%). PC2 was mostly associated with long elliptic, obovate, ovate, and round root shape (RS); cream, dark cream, and white predominant root flesh color (PRFC); cream and white secondary root flesh color (SRFC); deep green and light green leaf color (LC); and low, medium, and very high canopy coverage (CC). Elliptic, ovate, round, and round elliptic root shape (RS); cream, pale yellow, strongly pigmented, and white predominant root flesh color (PRFC); cream, dark cream, and white secondary root flesh color (SRFC); all subclasses of leaf color (LC) and canopy coverage (CC) were the key traits accounted for the third PC. The fourth PC was mainly associated with elliptic, oblong, obovate, round, and round elliptic root shape (RS); cream, pale yellow, strongly pigmented, and white predominant root flesh color (PRFC); all subclasses of secondary root flesh color (SRFC); deep green and light green leaf color (LC); and low, medium, and very high canopy coverage (CC). The fifth PC was mainly related to the traits round and round elliptic root shape (RS); cream, dark cream, pale yellow, and strongly pigmented predominant root flesh color (PRFC); dark cream, pale yellow, and white secondary root flesh color (SRFC); deep green and light green leaf color (LC); and all subclasses of canopy coverage (CC).

#### 2.3.2. Principal Component Analysis of the Quantitative Agro-Morphological and Physiological Traits

To assess the patterns of variation among the 26 agro-morphological and physiological quantitative traits, principal component analysis (PCA) was performed. The first nine principal components (PCs) accounted for a cumulative variance of 69.96% of the total variation observed between the accessions (Table 5). The PCA revealed that the source of variation was distributed across several components. Specifically, the first principal component (PC1) contributed to 20.27% of the variance and was primarily influenced by traits such as leaf length, leaf diameter, leaf area, the root length-to-diameter ratio, root yield, and root weight, all of which had relatively large positive weights on this component. The second (PC2), third (PC3), fourth (PC4), and fifth (PC5) principal components contributed 9.33%, 7.32%, 6.95%, and 6.35% to the total variation, respectively. The traits responsible for the variation in these components include fruit length, fruit diameter, fruit length-to-diameter ratio, fruit weight, the number of seeds per fruit, and the number of seeds per locule in PC2. In PC3, the key traits were petiole length, internode length, root yield, root weight per plot, and leaf area. For PC4, the contributing traits included root length, root diameter, root number per plot, leaf area index, gap fraction leaf area index, and normalized difference vegetative index. Lastly, the fifth PC was influenced by canopy density, fruit length, gap fraction leaf area index, and root number per plot. The sixth, seventh, eighth, and ninth PCs accounted for 5.89%, 4.94%, 4.67%, and 4.25% of the total variation, respectively. The traits contributing to these principal components included fruit diameter, fruit length-to-diameter ratio, and canopy density in PC6; petiole length, internode length, and the internode length to petiole length ratio in PC7; root length, root number per plot, and seed yield in PC8; and finally, internode length, thousand seed weight, and chlorophyll content associated with the ninth PC.

**Figure 5:**
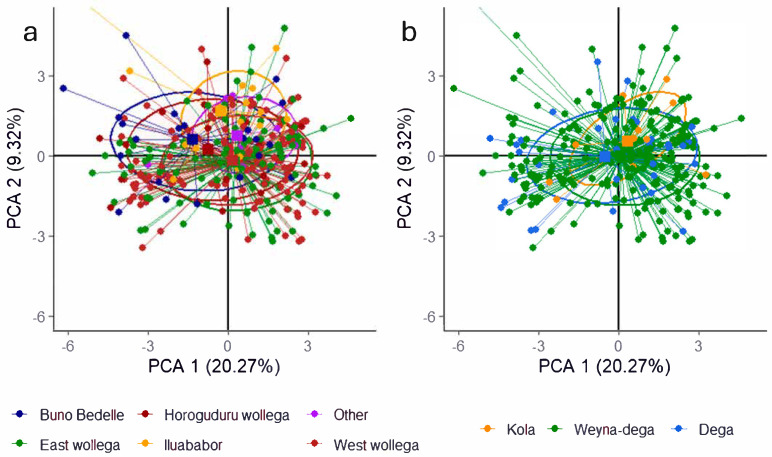
PCA plot indicating the relationship between the 26 quantitative agro-morphological and physiological traits across the geographic growing zones (**a**), and altitude (**b**).

The PC plot analysis for the distribution of accessions across collection zones and altitude levels showed a small variability. The plot showing distribution of accessions around collection zones indicates a slight separation for Buno Bedelle and Horoguduru wollega. This suggests there are some differences in the underlying variables that separate them from the rest, but it is not a complete separation. Mostly, many ellipses highly overlap, which indicates that there is no very strong separation between many of these zones for these first two principal components (Figure 5).

#### 2.3.3. Inter-Trait Relatedness of Qualitative and Quantitative Agro-Morphological and Physiological Traits

**Figure 6:**
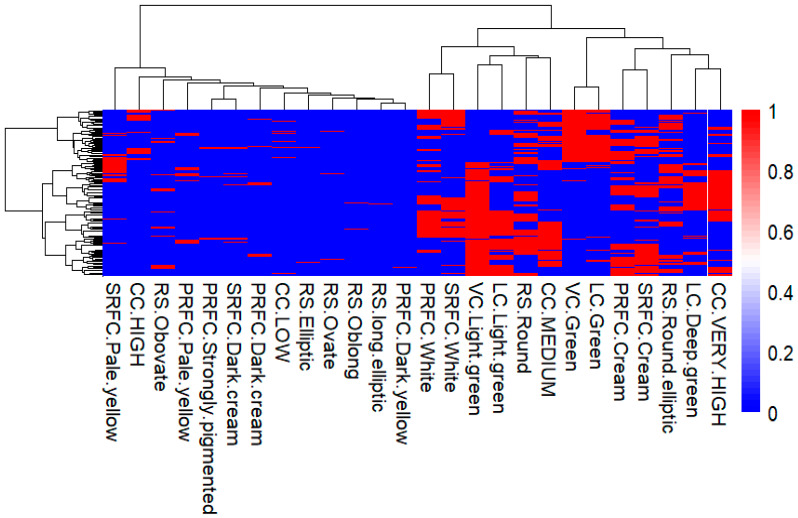
Heatmap showing hierarchical cluster analysis of 282 anchote accessions using Ward’s d2 into different clusters for six qualitative morphological traits.

As it shows in the heatmap, the hierarchical cluster analysis categorized anchote accessions into two distinct clusters based on six qualitative morphological traits (Figure 6). Cluster I included 194 accessions, accounting for 68.8%, while Cluster II comprised 88 accessions, making up 31.2%. Cluster I consists of accessions collected from the following locations: Bench Maji (1), Buno Bedelle (13), East wollega (74), Horro Guduru wollega (13), Iluababor (12), Jimma (1), Qelem wollega (1), West Shoa (1), and West wollega (78). In contrast, Cluster II includes accessions from: Buno Bedelle (9), East Gojam (1), East wollega (24), Horro Guduru wollega (3), Iluababor (4), Jimma (1), Qelem wollega (1), and West wollega (45) (Table 6). The dominant traits in Cluster I are light green vine color, light green leaf color, very high canopy coverage, and round root shape. In Cluster II, the predominant traits are green vine and leaf color. The traits with dominant red color indicate the dominance in that cluster, whereas traits with blue color showed no dominance (Figure 6).

**Figure 7:**
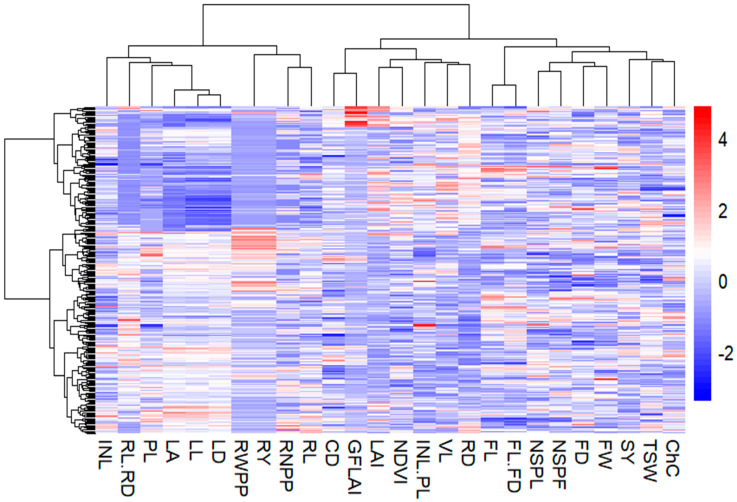
Heatmap showing hierarchical cluster analysis of 282 accessions of anchote using Ward’s d2 into different clusters for the 26 quantitative agro-morphological and physiological traits.

Heatmap clustering of the 282 anchote accessions was conducted based on 26 quantitative agro-morphological and physiological traits (Figure 7). The accessions were divided into three clusters with similar characteristics depending on the variables assessed. These distinctive clusters were formed according to the diversity of the recorded qualitative morphological traits. Cluster I, the largest cluster, included 122 accessions collected from various regions: Bench Madji (1), Buno Bedelle (6), East wollega (41), Horoguduru wollega (5), Iluababor (10), Jimma (2), Qelem wollega (1), West Shoa (1), and West wollega (55). This cluster represents 43.26% of the total accessions evaluated. The second cluster, which contained the fewest accessions, consisted of 55 entries, accounting for 19.5% of the total. These were collected from Buno Bedelle (2), East wollega (24), Horoguduru wollega (3), Iluababor (1), and West wollega (25). The third cluster comprised 105 accessions from Buno Bedelle (14), East Gojam (1), East wollega (33), Horoguduru wollega (8), Iluababor (5), Qelem wollega (1), and West wollega (43), making up 37.23% of the total accessions. The clustering results indicate that the pattern of cluster association is not much influenced by the geographical collection areas.

The accessions in the first cluster were defined by their highest mean values for petiole length, leaf diameter, fruit length, fruit diameter, fruit length-to-diameter ratio, thousand seed weight, seed yield, and chlorophyll content (Table 6). The second cluster was defined by leaf length, internode length, root number per plot, root length, root length-to-diameter ratio, root weight per plot, root yield, and leaf area, whereas the highest mean value of vine length, internode length to petiole length ratio, number of seeds per locule, number of seeds per fruit, fruit weight, root diameter, leaf area index, canopy density, gap fraction leaf area index, and normalized difference vegetative index were characterized in Cluster III.

**Figure 8:**
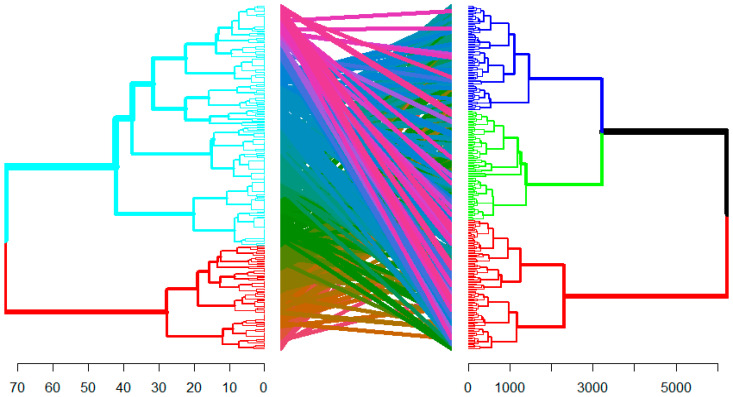
Tanglegram compares dendrograms based on evaluation of 282 anchote accessions using qualitative (**left**) and quantitative data (**right**), morpho-physiological, and agronomic data. Different coloured lines connect matching labels between the two dendrograms.

After the separate clustering of anchote accessions using qualitative and quantitative traits, a tanglegram was plotted to compare the relationship of these two clusters. The comparison indicated that 34.75% of the accessions preserved their cluster membership in the qualitative and quantitative agro-morphological and physiological clustering (Figure 8). The mantel statistics based on Pearson’s product-moment correlation also showed a significance value of 0.001, which indicates these two clusters can be entangled.

## 3. Discussion

### 3.1. Agro-Morphological and Physiological Traits Variation Among Anchote Accessions

This comprehensive investigation on anchote accessions using quantitative and qualitative agro-morphological and physiological traits, coupled with a thorough multivariate analysis, provided a greater insight into the existence of wider diversity and agronomic values.

The results revealed the presence of a significant morphological variation among anchote accessions in root shape, predominant and secondary root flesh color, vine and leaf color, and canopy coverage. Round and round elliptic root shapes were sorted out from the assessment as the major root shapes of anchote with a wider range of variability among the accessions, indicating an opportunity for improvement strategies. Although creamy and white flesh color dominated the primary and secondary root flesh color categories, accessions with highly pigmented flesh color showed the presence of natural anthocyanins and flavonoids. The development of these pigments has been widely indicated in yam [31] and sweet potato [32] and is linked to quality nutrition and health benefits for food and feed. Canopy coverage is an important trait influencing light interception, hence, affecting the photosynthetic rate. The presence of accessions with very high to medium canopy coverage indicates that the majority of the accessions have a strong spreading ability, which is beneficial for enhanced photosynthesis, and the leaves can be used for animal feed. Overall, the observed qualitative variations in these traits suggest that anchote has a considerable genetic diversity for these characters that could be used in future improvement programs for desirable agronomic characteristics such as increased nutritional quality, enhanced photosynthesis, and dual purposes of food and feed.

Similarly, the studied quantitative agro-morphological and physiological traits showed significant variation. The study revealed most of the traits, including all root traits, i.e., the yield part measured, showed wider ranges. Similar results were reported by [28]. This has significant implications, as yield is one of the vital agronomic and economic traits of such crops. The presence of variability in terms of their area of collection and altitude will help in selecting accessions for future crossings based on their place of origin and root traits.

In a similar pattern, the high values of PCV (phenotypic coefficient of variation) and GCV (genotypic coefficient of variation) for certain traits, such as leaf characteristics (length, diameter, area, and area index), vine length, seed yield, root number, and root yield, indicate that these traits are affected by both genetic and environmental factors. Accordingly, traits with high PCV and GCV values suggest significant genetic variability contributing to the total variability. Other studies with the same findings were reported by [33] in sweet potato genotypes and by [34] in yam genotypes. On the other hand, traits exhibiting the lowest GCV and moderate PCV indicate limited potential for improvement due to low genetic variability, suggesting that environmental factors have a moderate influence on the performance of these traits. Notably, the PCV values were greater than the GCV values for all characters, indicating these traits can contribute to future anchote improvement works.

Heritability is the proportion of phenotypic traits or total variance that is inherited from the parents [35]. The broad-sense heritability estimates observed for most of the agro-morphological traits in anchote fall within a moderate to high range, indicating the presence of sufficient genetic variation for effective selection. This is comparable to heritability values reported in white guinea yam and taro, which are moderate to high values [36,37]. Highly heritable traits like root yield can be aligned with farmer-preferred traits such as root shape by first fixing high-yielding lines, and then crossing them with genotypes having the desired shape, followed by multi-environment evaluation of the resulting progenies. A low broad-sense heritability value for the number of seeds per locule in this study indicates that the genetic contribution to the observed variation is small compared with the environmental variance. Environmental factors likely contributed significantly to the phenotypic variance and reduced the heritability estimate. Selection based on these traits would slow the genetic gain and reduce selection accuracy and breeding progress. Moderate to high heritability of physiological traits like chlorophyll content, NDVI, and leaf area index supports their use in indirect selection for stress tolerance by enhancing alleles that maintain greenness and photosynthetic efficiency under heat or drought, accelerating the breeding of climate-resilient varieties.

The presence of high genetic advance as a percentage of the mean coupled with high heritability for root yield, root weight, seed yield, vine length, and leaf area index imply the selection of these traits is a reliable strategy for breeding of anchote genotypes. Although the estimates of heritability provide information about the extent of the transfer of quantitative traits, if coupled with genetic advance, it presents a better measure of genetic gain [34,38]. This indicates that variability is a result of an additive gene effect. Where additive genes are less affected by environment, phenotypic selection might be impactful for improving these quantitative traits, including the physiological characteristics [35,39,40,41].

### 3.2. Inter-Trait Relationship and Divergence

The significant and positive values of phenotypic correlations among quantitative traits indicate that the positive selection of one of the correlated traits leads to an increase in the other trait and vice versa. The result showed a significant positive correlation between agronomically important traits such as root yield and root diameter as well as root yield and leaf area. Traits with high correlation are among the key traits often considered important for the genotype selection in genetic improvement [9,28]. This showed that selection for root yield based on these traits will be a good strategy during selection for breeding divergently. Root yield had a significant negative correlation with vine length, internode length to petiole length ratio, fruit weight, number of seeds per fruit, and fruit diameter. This could be due to competing growth demands from the traits as a sink to the photosynthates. The negative correlation between root yield and vine length likely suggests a resource allocation trade-off (e.g., dry matter partitioning) between vegetative growth and storage organ development. In many root and tuber crops, such trade-offs are well documented. Similar results have been reported on cassava and sweet potato genotypes [42,43]

The principal component analysis revealed root traits such as root shape and color are key contributors to anchote accessions, indicating the presence of high diversity in storage organ morphology, environmental adaptation, and farmer preferences. In the study on the production potential of anchote in East Wollega, ref. [44] found that most farmers prefer a round root shape. The dominance of the root shape, root flesh color, and canopy coverage in the first two PCs also indicates that these traits are preferably selected by farmers, which can be useful for future breeding work. Variations in vine and leaf color may affect photosynthetic efficiency and stress tolerance. Additionally, canopy coverage can indicate plant vigor and competition for resources. Differences in the color of the root flesh, which are linked to pigment composition, suggest opportunities for nutritional enhancement and biofortification. Variations in root shape may also influence processing characteristics and market demand. Accessions with greater canopy coverage could be more suitable for resource-limited environments, making them ideal for low-input farming systems. These outcomes are aligned with the finding reported by [19].

The first principal component (PC1) share accounts for over one-fifth of the total variation, comprising both above- and below-ground growth, including leaf and root characteristics such as leaf length, leaf diameter, leaf area, root length-to-diameter ratio, root yield, and root weight. This suggests the variation captured in PC1 highlights a potential trait correlation between canopy vigor, stress resilience, and root yield. Ref. [44] also identified leaf length and root yield as the most significant traits in the initial principal components of anchote accessions. This information can be crucial for breeding programs focused on enhancing overall plant biomass and stress adaptation through optimized root architecture. The second principal component (PC2) explains an additional 9.33% of the variation and is mainly associated with fruit morphology and reproductive attributes. This indicates that variation along PC2 is largely determined by differences in fruit size and shape as well as reproductive potential, measured by seed count. These attributes are directly linked to yield quality and quantity, making PC2 an important factor when the breeding objective is to enhance fruit productivity for seed multiplication purposes. Traits such as petiole length, internode length, root yield, root weight per plot, and leaf area are central to the third principal component (PC3). The significance of root traits in this component emphasizes their critical role in overall variation, as they are also major determinants in PC1. In summary, the first three principal components collectively capture key dimensions of plant morphology, ranging from vegetative growth and root development (PC1), to fruit-related reproductive characteristics (PC2), and to integrated shoot and root architecture (PC3). The influence of different traits across these components highlights the complexity of phenotypic variation and offers insights for selecting traits in breeding programs aimed at improving overall plant performance.

The PCA plot indicates that neither the collection zone nor the altitude level provided a clear separation of accessions based on quantitative traits. This suggests that the variation captured by these traits is not strongly structured by these geographic or altitudinal factors. It is possible that other factors like the breeding nature of the crop, farmers’ selection, genetic background, gene flow, seed exchange among farmers, or similar selection pressures across regions have a greater impact on the observed variation. It may also reflect the limited impact of geographic isolation on trait differentiation. While collection zone and altitude might be expected to influence trait distribution [45] the overall nature of the crop should also be considered. This finding has significant implications for better differentiation and selection of accessions with desirable traits.

Clustering is an important method for studying associations among closely or distantly related genotypes by grouping units based on their similarity or dissimilarity in specific attributes or response patterns [46]. In population profiling and screening for breeding, clustering is important for selecting individuals with desirable traits for further breeding studies, as the genetic relationships of crop species are important in working on a specific set of breeding populations [47].

The cluster analysis of the six qualitative traits and their subclasses categorized the accessions into two separate clusters. The distinct dominant traits in each cluster suggest potential differences in adaptation and selection priorities. In the first cluster (C1), light green vine and leaf colors along with very high canopy coverage and round root shape are the dominant traits, which may indicate accessions optimized for vigorous growth, weed suppression, improved photosynthetic capacity, and better resource utilization, which can be crucial in competitive and variable environments. Round root shape could be associated with farmers’ preference and marketability, as was reported by [44]. High canopy coverage, for example, might contribute to improved photosynthetic capacity and better resource utilization, which can be crucial in competitive or variable environments. Cluster II is primarily characterized by green vine and leaf colors, which is also important considering the photosynthetic ability of accessions. In this group, the uniformity in color could imply a more conservative or stable phenotype under the evaluated conditions. This might be beneficial for breeding programs. In contrast, ref. [19] reported the grouping of anchote accessions using qualitative traits in six clusters, and the majority of the accessions were grouped in one cluster.

Similarly, clustering based on the 26 quantitative agro-morphological and physiological traits resulted in three groups. Cluster I is mainly characterized by accessions with better vegetative performance, while accessions in the second cluster are characterized by traits related to high root yield. The last cluster is also characterized mainly by a high value of physiological traits. These groupings enable a breeder to select accessions with a specific trait from each group and create a more comprehensive variety. Each cluster harbors accessions from different collection areas and agroecology. This might be due to seed exchange, genetic drift, and natural and artificial selection. It also indicates that key traits are widely distributed and not confined to specific locations. This indicates the broad adaptability of accessions across environments, making them valuable for breeding programs targeting wide agroecological zones. It also highlights the importance of conserving genetic diversity across all regions, as useful traits may occur independently of geographic origin. It has been shown in previous studies in some species such as Amaranthus and Black cumin that genetic diversity is not always directly connected to geographic origin or distribution of the accessions [22,48,49]. In these studies, a similar pattern was observed in the PCA biplot. Each cluster showed a unique trait profile, providing a clear framework for selecting accessions that match specific breeding objectives, whether that be yield improvement, enhanced stress tolerance, or better reproductive performance. The minimal influence of environmental factors such as altitude and collection zones on trait variation is also likely due to the single test environment masking genotype × environment (G × E) interactions, as many traits are environment sensitive. Multi-location trials are essential to reveal G × E effects, identify stable genotypes, and ensure accurate selection for breeding.

Lastly, the pattern in the tanglegram implies that while some accessions share similarities across both qualitative and quantitative agro-morphological and physiological trait categories, many of them are grouped differently. The 34.75% entanglement shows low similarity between qualitative and quantitative trait clustering, suggesting they are not strongly correlated. This might indicate the presence of low pleiotropy, and the traits are independently controlled for most of the accessions. Hence during selection, full consideration needs to be given to both qualitative and quantitative traits for effective breeding. Otherwise, relying on one trait type may not fully capture the genetic variation. Furthermore, the presence of low congruence and low pleiotropy is generally favored, as it allows for more targeted selection and trait improvement.

## 4. Materials and Methods

### 4.1. Materials Used

**Figure 9:**
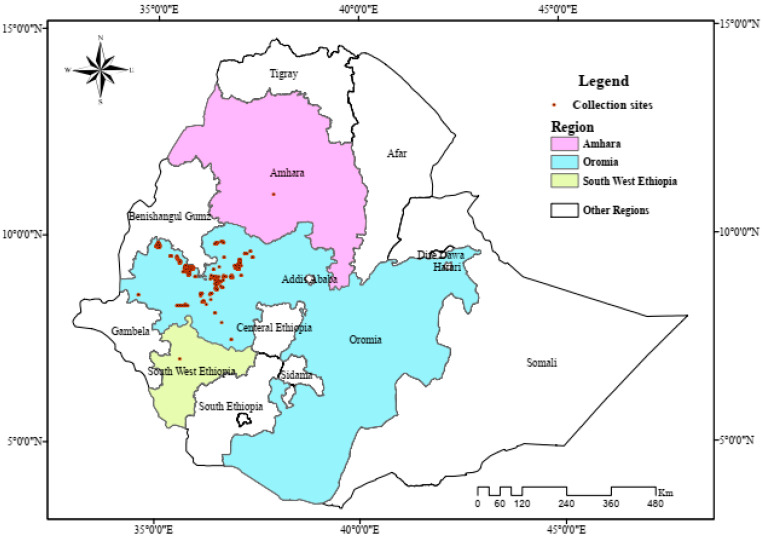
Map indicating the collection zones for various anchote accessions across different regions of Ethiopia.

Seeds from 282 anchote accessions and two checks, i.e., a standard check variety and a promising genotype in a breeding pipeline, were sourced from Debre Zeit Agricultural Research Center (DZARC), Bishoftu, Ethiopia. The accessions were initially collected from various geographical locations of Ethiopia, specifically Western, Southwestern, and Northwestern Ethiopia, encompassing 10 administrative zones and 40 administrative districts (Figure 9). The altitudinal range of the collection sites for the accessions was between 1412 and 3025 m above sea level (m.a.s.l). The highest number of accessions was collected from West Wollega and the least was from the West Shoa, Bench Madji, and East Gojam zones. The passport data of these accessions is shown in Table S3.

### 4.2. Description of the Study Area

The field evaluation trial was implemented at the DZARC experimental site located in Bishoftu, Ethiopia, with geographical coordinates of 08°44′ N latitude and 38°58′ E longitude. The site is at an altitude of 1860 m.a.s.l. This area experiences a mean annual rainfall of 851 mm, with average minimum and maximum temperatures of 8.9 °C and 24.3 °C, respectively. The soil at this site is classified as heavy black vertisols [50]

### 4.3. Experimental Design and Field Management

The field experiment was implemented using an Augmented Randomized Block Design with 14 blocks. Each accession was represented in one of the blocks randomly, and the two check varieties were replicated across all blocks to allow an error estimate from these replicated checks, which was used in the analysis to evaluate the remaining accessions. Furthermore, these replicated checks were used for the adjustment of between-block variability. To account for the variation that might come from soil heterogeneity, the blocks were arranged against the soil gradient, and checks were replicated across the blocks. Each experimental unit comprised two ridges per plot per accession with 0.6 m and 0.2 m spacing between rows and columns, respectively, and ten plants per plot. Each block had 22 plots.

The model for the design is as follows:

Y_ij_ = μ + B_j_ + T_i_ + ϵ_ij_

- Y_ij_: observed value of the response in block j, treatment (or genotype) i;
- μ: overall mean;
- B_j_: fixed effect of block j;
- T_i_: fixed effect of treatment i (includes both checks and unreplicated test entries);
- ϵ_ij_: random error term.

Seeds of the accessions were planted during the main cropping season in April 2023 in two rows on the ridge. During growth, the experimental field received supplemental irrigation as needed. Necessary agronomic practices such as weeding, earthing-up, and stalking were applied uniformly according to the recommendations for anchote.

### 4.4. Data Collection

#### Agro-Morphological and Physiological Traits

Data on quantitative, physiological, and qualitative agro-morphological traits were collected from five randomly selected accessions per plot, and the average was used for the data analysis. Since no established quantitative descriptors exist for anchote identification, trait descriptions for the current study were adopted and/or modified based on descriptors developed for *Cucurbita* spp. (particularly cucumber, melon, and watermelon) [51,52] along with some root and fruit descriptors unique to anchote, as described by [9,28].

Six qualitative characteristics, including root shape (RS), predominant root flesh color (PRFC), secondary root flesh color (SRFC), vine color (VC), leaf color (LC), and canopy coverage (CC) were recorded. All qualitative traits are recorded at the crop maturity stage. On the other hand, 26 quantitative traits, including petiole length (cm), leaf length (cm), leaf diameter (cm), internode length (cm), vine length (cm), internode length to petiole length ratio (cm), fruit length (cm), fruit diameter (cm), fruit length-to-diameter ratio (cm), number of seeds per locule, NSPF = number of seeds per fruit, FW = fruit weight (g), TSW = thousand seed weight (g), SY = seed yield (g), RNPP = root number per plot, RL = root length (cm), RD = root diameter (cm), RL/RD = root length-to-diameter ratio (cm), RWPP = root weight per plot (kg), RY = root yield (t/ha), LA = leaf area (cm^2^), LAI = leaf area index, CD = canopy density, GFLAI = gap fraction leaf area index, CC = chlorophyll content, and NDVI = normalized difference vegetative index (%) were recorded (Supplementary Tables S1 and S2).

### 4.5. Data Analysis

Both qualitative and quantitative agro-morphological and physiological traits were used to assess the diversity of the anchote accessions. All statistical analyses were performed using R software version 4.4.1 [53]. To estimate the extent of qualitative morpho-agronomic diversity of anchote accessions, the frequency of qualitative trait class and Shannon–Weaver diversity index (*H′*) were calculated. Shannon–Weaver diversity index (*H′*) estimation and chi-square (X^2^) analysis were performed using R vegan 2.6.8 package [54]. The index, according to [55], was calculated as

$$H^{\prime }=-\sum_{\left\{i=1\right\}}^{\left\{n\right\}}pilnpi$$

where *n* = number of categories, *pi* = proportion of individuals in the *i*th category, *lnpi* = natural logarithm of *pi*.

To estimate quantitative trait variations, analysis of variance (ANOVA) was performed along with mean; standard error; range; the estimates of variance components including phenotypic coefficient of variation (PCV), genotypic coefficient of variation (GCV), broad-sense heritability (Hb), and genetic advance (GA); and genetic advance as percentage of the mean (GAM). The analysis was carried out using the augmented RCBD 0.1.7 package [56]. To assess the magnitude and direction of the measured quantitative morpho-agronomic traits, a phenotypic correlation analysis was conducted using the ‘corrplot 0.94′ and ‘metan 1.19.0’ package [57,58]. For principal component and cluster analysis of qualitative traits, the data was changed to numeric using the one-hot encoding method. Principal component analysis (PCA) for both qualitative and quantitative traits was performed by factoextra 1.0.7, MASS 7.3.61, and ggplot2 3.5.1 package [59,60,61]. Cluster analysis of qualitative traits was performed using cluster 2.1.8, factoextra 1.0.7, dplyr 1.1.4, and ggplot2 3.5.1 package of R software [59,61,62,63]. This was carried out using Gower’s distance [64] and Ward.D² [65] method. Optimum number of clusters of qualitative traits was determined using silhouette method [66]. Cluster analysis with complete linkage method for quantitative traits was carried out to categorize entries of germplasm collections based on the degree of similarity and dissimilarity [67] Hierarchical clustering following ward D2 approach was conducted using factoextra 1.0.7, cluster 2.1.8, and pheatmap 1.0.12 packages of R software [59,62,68]. The Mantel test was performed to test the correlation of qualitative and quantitative traits distance matrix [69]. After this test, tanglegram, which connects and compares the relation of clusters plotted for qualitative and quantitative traits, was plotted using dendextend 1.17.1 package [70].

## 5. Conclusions

Phenotypic evaluation coupled with multivariate analysis was a powerful tool in profiling the anchote accessions. The study was able to display the presence of wider variation between the anchote accessions based on qualitative and quantitative agro-morphological and physiological traits. While some phenotypic classes of given qualitative and quantitative features were unique in particular zones and altitudes, others were found to be widespread across all zones and altitudes. Root yield, seed yield, vine length, leaf area index, and gap fraction leaf area index presented high heritability along with genetic advance as a percentage of mean and significant inter-trait relation with other quantitative traits. These traits should be prioritized in breeding programs, as they are largely governed by additive gene action and respond well to selection. Color- and shape-related qualitative traits of roots, color-related traits of vine and leaf, and canopy coverage highly contributed to the exhibited variability as the most influential traits. Cluster analysis based on both qualitative and quantitative traits grouped the accession into two and three main clusters with distinct trait patterns, respectively, which are not connected to geographic origin. Taken together, these traits can be used as selection markers to facilitate breeding strategies of anchote. As the future direction, the nutritional characterization of the accessions is crucial to unlock their potential for developing nutrient-rich varieties.

## Figures and Tables

**Table 1:** Specific qualitative morphological traits, their scores, equivalent phenetic characters, (%) contribution to variation, and chi-square for six morphological qualitative traits. Statistical significance * *p* < 0.05, and *** *p* < 0.001.

Qualitative Marker	Character State	Proportion	% Proportion	Chi-Square
Root shape (RS)	Elliptic	7	2.48227	456.77 ***
	Long elliptic	2	0.70922	
	Oblong	4	1.41844	
	Obovate	24	8.510638	
	Ovate	4	1.41844	
	Round	126	44.68085	
	Round elliptic	115	40.78014	
Predominant root flesh color (PRFC)	Cream	124	43.97163	331.95 ***
	Dark cream	13	4.609929	
	Dark yellow	1	0.35461	
	Pale yellow	21	7.446809	
	Strongly pigmented	10	3.546099	
	White	113	40.07092	
Secondary Root flesh color (SRFC)	Cream	123	43.61702	103.56 ***
	Dark cream	12	4.255319	
	Pale yellow	50	17.7305	
	White	97	34.39716	
Vine color (VC)	Green	92	32.62411	34.05 ***
	Light green	190	67.37589	
Leaf color (LC)	Deep green	75	26.59574	9.08 *
	Green	91	32.2695	
	Light green	116	41.13475	
Canopy coverage (CC)	High	29	10.28369	153.57 ***
	Low	9	3.191489	
	Medium	125	44.32624	
	Very high	119	42.19858	

**Table 2:** Descriptive statistics and coefficient of variation of 26 quantitative agro-morphological and physiological traits of 282 evaluated anchote accessions.

Traits	Mean	SE	Minimum	Acc (min)	Maximum	Acc (max)	CV
Petiole length (PL)	3.72	0.09	1.00	Acc.202	7.60	Acc.19	30.09
Leaf length (LL)	6.54	0.12	1.00	Acc.236	10.67	Acc.120	19.62
Leaf diameter (LD)	6.69	0.12	1.10	Acc.236	11.00	Acc.379	19.34
Internode length (INL)	10.25	0.11	5.50	Acc.151	15.20	Acc.124	12.30
Vine length (VL)	2.16	0.04	1.00	Acc.52	3.90	Acc.145	19.93
Internode length to petiole length ratio (INL/PL)	2.98	0.07	1.05	Acc.19	7.33	Acc.3	24.35
Fruit length (FL)	5.32	0.06	3.20	Acc.170	8.60	Acc.348	12.98
Fruit diameter (FD)	4.12	0.03	2.80	Acc.50	5.80	Acc.117	9.84
Fruit length-to-diameter ratio (FL/FD)	1.30	0.01	0.74	Acc.271	2.00	Acc.363	13.11
Number of seeds per locule (NSPL)	17.99	0.22	10.40	Acc.7	29.40	Acc.363	16.54
Number of seeds per fruit (NSPF)	102.55	1.41	50.00	Acc.85	166.60	Acc.363	13.64
Fruit weight (FW)	328.37	5.95	164.41	Acc.51	678.20	Acc.348	18.96
Thousand seed weight (TSW)	45.20	0.66	21.10	Acc.146	71.20	Acc.117	18.85
Seed yield (SY)	463.23	14.53	99.80	Acc.160	973.80	Acc.412	31.18
Root number per plot (RNPP)	1.98	0.06	1.00	Acc.7	5.00	D-01	37.55
Root length (RL)	11.70	0.14	7.32	Acc.363	17.50	D-01	17.99
Root diameter (RD)	8.07	0.21	2.50	Acc.57	16.44	Acc.200	25.76
Root length-to-diameter ratio (RL/RD)	1.61	0.05	0.91	Acc.345	3.75	Acc.241	29.66
Root weight per plot (RWPP)	1.79	0.10	0.17	Acc.68	7.00	Acc.129	50.80
Root yield (RY)	59.67	3.40	5.57	Acc.68	233.33	Acc.129	50.80
Leaf area (LA)	30.93	1.11	0.53	Acc.334	81.6	Acc.379	38.83
Leaf area index (LAI)	0.91	0.05	0.100	Acc.6	2.90	Acc.262	42.81
Canopy density (CD)	37.33	0.51	13.20	Acc.68	59.9	Acc.207	14.03
Gap fraction leaf area index (GFLAI)	0.57	0.02	0.100	Acc.57	2.40	Acc.278	15.77
Chlorophyl content (ChC)	52.66	0.64	24.30	Acc.291	79.7	Acc.57	15.68
Normalized difference vegetative index (NDVI)	59.35	0.85	27.00	Acc.224	87.0	Acc.105	11.52

**Table 3:** Genetic variability, heritability, and genetic advance for quantitative agro-morphological and physiological traits in anchote accessions.

Traits	Mean	PCV (%)	GCV (%)	Hb^2^ %	GA	GAM %
Petiole length (PL)	3.70	30.49				
Leaf length (LL)	6.51	30.15	22.67	56.54	2.29	35.17
Leaf diameter (LD)	6.67	30.07	22.89	57.93	2.40	35.93
Internode length (INL)	10.20	14.75	8.21	30.99	0.96	9.43
Vine length (VL)	2.15	32.20	25.48	62.62	0.90	41.60
Internode length to petiole length ratio (INL/PL)	2.98	30.22	18.52	37.54	0.70	23.40
Fruit length (FL)	5.30	18.29	12.69	48.14	0.96	18.17
Fruit diameter (FD)	4.11	11.49	5.83	25.76	0.25	6.11
Fruit length-to-diameter ratio (FL/FD)	1.30	17.17	10.97	40.83	0.19	14.47
Number of seeds per locule (NSPL)	17.87	17.96	6.69	13.85	0.92	5.13
Number of seeds per fruit (NSPF)	102.66	18.09	11.80	42.57	16.31	15.88
Fruit weight (FW)	327.85	23.25	13.06	31.52	49.58	15.12
Thousand seed weight (TSW)	45.37	22.76	13.10	33.11	7.05	15.55
Seed yield (SY)	462.67	49.73	38.68	60.49	287.13	62.06
Root number per plot (RNPP)	1.97	43.77	21.13	23.30	0.41	21.04
Root length (RL)	11.62	15.90				
Root diameter (RD)	7.96	31.82	18.27	32.97	1.72	21.64
Root length-to-diameter ratio (RL/RD)	1.62	36.04	20.35	31.87	0.38	23.70
Root weight per plot (RWPP)	1.79	92.76	77.65	70.06	2.40	134.08
Root yield (RY)	59.77	92.76	77.6	70.06	80.13	134.08
Leaf area (LA)	30.93	59.56	44.65	56.20	21.51	69.06
Leaf area index (LAI)	0.91	79.60	68.58	74.22	1.14	121.89
Canopy density (CD)	37.33	19.29	13.23	47.03	6.98	18.71
Gap fraction leaf area index (GFLAI)	0.57	68.85	67.13	95.05	0.78	135.01
Chlorophyl content (ChC)	52.66	19.34	11.29	34.08	7.16	13.60
Normalized difference vegetative index (NDVI)	59.35	21.86	18.49	71.58	19.16	32.28

GCV = genotypic coefficients of variation, PCV = phenotypic coefficients of variation, Hb^2^ = broad-sense heritability, GA = genetic advance (in the unit of each trait), GAM = genetic advance as percentage of the mean.

**Table 4:** Eigenvectors and eigenvalues of the first 9 principal components for 6 qualitative morphological characters of 282 anchote accessions.

	PC1	PC2	PC3	PC4	PC5	PC6	PC7	PC8	PC9	PC 10
RS.Elliptic	0.08	0.00	2.57	3.57	0.04	0.00	0.98	12.95	0.19	1.16
RS.long.elliptic	0.14	1.34	0.22	0.03	0.03	0.42	0.44	1.97	0.93	2.82
RS.Oblong	0.01	0.38	0.01	0.91	0.18	1.45	0.04	1.58	0.01	3.32
RS.Obovate	0.64	0.55	0.25	1.30	0.01	0.57	25.17	2.86	21.86	0.24
RS.Ovate	0.01	1.05	1.37	0.06	0.00	0.08	2.74	3.57	5.84	35.58
RS.Round	0.11	0.72	4.65	7.56	15.64	17.85	7.59	1.13	0.04	2.08
RS.Round.elliptic	0.58	0.29	6.95	9.19	15.52	12.24	0.88	4.09	3.49	0.01
PRFC.Cream	0.03	23.21	0.49	1.82	0.79	0.02	0.46	3.72	9.61	8.42
PRFC.Dark.cream	0.16	0.68	0.04	0.06	0.48	1.59	12.41	6.61	6.46	28.27
PRFC.Dark.yellow	0.18	0.14	0.00	0.11	0.01	0.15	16.72	3.51	18.63	0.05
PRFC.Pale.yellow	0.05	0.15	4.95	9.77	4.35	11.89	0.16	0.07	4.68	0.82
PRFC.Strongly. pigmented	0.31	0.00	9.42	8.64	12.22	11.27	0.02	0.32	0.06	2.94
PRFC.White	0.10	25.84	2.41	2.19	0.15	0.02	0.24	0.31	0.22	0.64
SRFC.Cream	0.30	19.13	0.48	12.28	0.13	1.37	0.24	2.49	0.65	1.02
SRFC.Dark.cream	0.04	0.01	11.52	13.86	10.80	8.60	0.00	0.15	0.02	0.01
SRFC.Pale.yellow	0.00	0.00	0.01	14.35	9.43	17.43	0.99	0.01	2.42	0.79
SRFC.White	0.18	20.57	4.44	0.94	0.48	0.79	1.71	1.95	4.60	0.09
VC.Green	23.74	0.30	0.04	0.01	0.23	0.74	0.25	0.06	0.01	0.48
VC.Light.green	23.74	0.30	0.04	0.01	0.23	0.74	0.25	0.06	0.01	0.48
LC.Deep.green	6.12	0.69	9.00	1.43	5.05	0.00	4.63	18.38	1.84	0.34
LC.Green	21.25	0.23	0.83	0.18	0.00	0.05	0.28	0.05	1.36	0.02
LC.Light.green	4.66	1.46	12.65	0.44	4.17	0.03	5.94	16.50	5.41	0.15
CC.HIGH	10.34	0.00	0.96	0.14	1.44	0.04	0.10	2.38	0.28	2.11
CC.LOW	0.95	0.58	2.16	0.43	0.43	6.58	9.95	1.65	8.89	8.03
CC.MEDIUM	0.03	1.51	12.48	6.39	11.92	4.35	6.34	8.05	2.02	0.11
CC.VERY.HIGH	6.25	0.87	12.06	4.33	6.27	1.71	1.48	5.59	0.48	0.04
Eigenvalue	3.7	2.58	2.22	2	1.86	1.49	1.36	1.28	1.19	1.08
Proportion	14.22	9.92	8.56	7.68	7.16	5.73	5.25	4.92	4.59	4.17
Cumulative	14.22	24.14	32.7	40.38	47.54	53.27	58.52	63.44	68.03	72.2

**Table 5:** Eigenvalues and eigenvectors of the first nine principal components for quantitative agro-morphological and physiological traits of anchote accessions.

Traits	Principal Components								
	PC1	PC2	PC3	PC4	PC5	PC6	PC7	PC8	PC9
Eigenvalue	5.27	2.43	1.90	1.81	1.65	1.53	1.28	1.21	1.10
Proportion	20.27	9.33	7.32	6.95	6.35	5.89	4.94	4.67	4.25
Cumulative	20.27	29.60	36.92	43.87	50.21	56.11	61.05	65.72	69.96
**Eigenvectors**									
Petiole length	0.254	−0.047	0.335	−0.218	0.042	−0.020	−0.361	0.247	−0.093
Leaf length	0.370	−0.008	0.225	0.059	0.029	−0.030	0.187	0.024	0.062
Lea diameter	0.374	−0.036	0.205	0.076	0.029	−0.045	0.167	−0.029	0.074
Internode length	0.078	0.027	0.323	0.064	−0.010	−0.256	0.459	0.143	−0.289
Vine length	−0.230	0.091	0.015	−0.007	0.028	−0.034	0.101	0.327	−0.206
Internode length to petiole length ratio	−0.211	0.076	−0.211	0.231	−0.063	−0.087	0.603	−0.146	0.042
Fruit length	−0.046	−0.512	−0.088	0.011	0.255	−0.276	−0.028	−0.058	−0.109
Fruit diameter	−0.038	−0.319	0.128	−0.004	0.057	0.452	0.145	−0.063	0.054
Fruit length-to-diameter ratio	−0.023	−0.319	−0.175	0.017	0.230	−0.588	−0.120	−0.030	−0.156
Number of seeds per locule	−0.074	−0.351	0.099	−0.005	−0.219	0.018	0.091	0.187	0.238
Number of seeds per fruit	−0.086	−0.434	0.121	−0.013	−0.118	0.117	0.091	0.029	0.117
Fruit weight	−0.075	−0.375	0.041	−0.032	−0.020	0.162	0.091	0.141	0.048
Thousand seed weight	−0.019	−0.096	0.002	−0.020	−0.167	0.280	−0.060	−0.304	−0.462
Seed yield	−0.034	−0.172	−0.026	0.066	−0.141	0.083	−0.069	−0.361	−0.005
Root number per plot	0.130	−0.041	−0.057	−0.334	0.216	0.029	0.042	−0.349	0.018
Root length	0.197	0.025	0.022	−0.303	0.117	0.007	0.124	−0.301	−0.019
Root diameter	−0.245	0.102	0.247	−0.298	0.329	0.009	0.139	−0.215	0.007
Root length-to-diameter ratio	0.283	−0.072	−0.200	0.211	−0.288	−0.005	−0.098	0.057	−0.042
Root weight per plot	0.255	−0.041	−0.416	−0.294	−0.083	0.063	0.170	0.140	−0.094
Root yield	0.255	−0.041	−0.416	−0.294	−0.083	0.063	0.170	0.140	−0.094
Leaf area	0.371	−0.027	0.217	0.069	0.032	−0.034	0.180	−0.015	0.052
Leaf area index	−0.202	0.017	0.139	−0.280	−0.125	−0.003	0.073	0.180	−0.075
Canopy density	−0.042	0.011	0.125	−0.193	−0.465	−0.290	−0.013	−0.256	0.136
Gap fraction LAI	−0.094	0.004	0.115	−0.288	−0.484	−0.262	−0.011	−0.101	0.066
Chlorophyl content	0.010	−0.043	0.131	0.160	−0.163	0.052	−0.031	−0.099	−0.672
Normalized difference vegetative index	−0.128	−0.017	−0.001	−0.374	0.014	0.072	0.102	0.287	−0.163

**Table 6:** Mean value of 26 agro-morphological and physiological quantitative traits of 282 anchote accessions collected from Ethiopia in each cluster.

Traits	Clusters		
	Cluster I	Cluster II	Cluster III
Petiole length (cm)	4.061	4.054	3.173
Leaf length (cm)	7.448	7.624	4.919
Leaf diameter (cm)	7.743	7.684	4.984
Internode length (cm)	10.294	10.365	10.135
Vine length (m)	1.830	2.103	2.588
Internode length to petiole length ratio (cm)	2.705	2.822	3.363
Fruit length (cm)	5.437	5.075	5.298
Fruit diameter (cm)	4.142	4.027	4.129
Fruit length-to-diameter ratio (cm)	1.322	1.265	1.290
Number of seeds per locule	18.197	16.767	18.310
Number of seeds per fruit	104.203	94.037	104.688
Fruit weight (gm)	329.828	305.729	337.918
Thousand seed weight (gm)	47.040	42.904	44.358
Seed yield (gm)	475.791	419.218	471.594
Root number per plot	2.112	2.236	1.695
Root length (cm)	12.148	12.461	10.811
Root diameter (cm)	6.888	6.719	10.138
Root length-to-diameter ratio (cm)	1.910	1.918	1.101
Root weight per plot (kg)	1.603	4.331	0.689
Root yield (t)	53.449	144.351	22.960
Leaf area	40.916	40.934	15.033
Leaf area index	0.672	0.649	1.321
Canopy density	36.467	38.213	38.380
Gap fraction leaf area index	0.487	0.524	0.705
Chlorophyl content	53.671	52.575	51.793
Normalized difference vegetative index	55.670	59.436	64.029

## Data Availability

The datasets generated and/or analyzed during the current study are not publicly available, as they will be integrated with additional data for a subsequent publication. However, they are available from the corresponding author upon reasonable request.

## Supplementary Materials

The following supporting information can be downloaded at: https://www.mdpi.com/article/10.3390/plants14152334/s1, Table S1: list of Qualitative traits with their respective codes and description; Table S2: List of quantitative agro-morphological and physiological traits recorded; Table S3: Passport data of the accessions collected.

## Abbreviations

ANOVA	Analysis of variance
DZARC	Debre Zeit Agricultural Research Center
GA	Genetic advance
GAM	Genetic advance as a percentage of the mean
GCV	Genotypic coefficient of variation
PC	Principal component
PCA	Principal component analysis
PCV	Phenotypic coefficient of variation

## References

[1] Kenyon L., Anandajayasekeram P., Ochieng C. A Synthesis/Lesson-Learning Study of the Research Carried out on Root and Tuber Crops Commissioned Through the DFID RNRRS Research Programmes Between 1995 and 2005. A Report Submitted to the Crop Protection Program (CPP) of the UK Department for Inter. Natural Resources Institute & IFPRI-ISNAR, UK 2006. https://www.researchgate.net/publication/216088436.

[2] Habtamu F.G. (2014). Nutritional composition, antinutritional factors and effect of boiling on nutritional composition of Anchote (*Coccinia abyssinica*) tubers. J. Sci. Innov. Res..

[3] Tilahun W., Sentayehu A., Amsalu A., Weyessa G. (2014). Genetic diversity analysis among Anchote (*Coccinia abyssinica*) Accessions in Western Ethiopia. Int. J. Agric. Res..

[4] Tileye F. (2020). *Coccinia abyssinica* (lam.) Cogn. (anchote) biology, productivity, and prospects of genetic improvement using biotechnological tools. J. Hortic. Res..

[5] Holstein N., Renner S. (2011). A dated phylogeny and collection records reveal repeated biome shifts in the African genus Coccinia (Cucurbitaceae). BMC Evol. Biol..

[6] Getahun A. (1973). Developmental anatomy of tubers of Anchote: A potential dryland tuber crop. Acta Hort..

[7] Hassen Y., Ali M., Desta F., Seid H. (2013). Effect of Flower Bud Removal on Growth and Yield of Anchote Root (*Coccinia abyssinica* (Lam.) Cogn.) Accessions at Bishoftu. Adv. Res. J. Plant Anim. Sci..

[8] Abreham B., Tileye F., Kassahun T. (2014). Genetic diversity of anchote (*Coccinia abyssinica* (Lam.) Cogn.) from Ethiopia as revealed by ISSR markers. Genet. Resour. Crop Evol..

[9] Bekele S.T. Morphological and Molecular Genetic Diversity and Cytogenetics of Cultivated Anchote (*Coccinia abyssinica* (Lam.) Cogn.) from Ethiopia. A Dissertation Submitted to the Department of Microbial Cellular and Molecular Biology, School of Graduate Studies, Addis Ababa University 2017. https://etd.aau.edu.et/server/api/core/bitstreams/8b4b642f-a64f-4bb3-822a-0fe1a80ba456/content.

[10] Ayalew Y., Retta N., Mohammed A., Kim K.S., Kobue L., Haki R.I. (2019). Phytochemical constituents in edible parts of anchote (*Coccinia abyssinica* (lam.) (cogn.)) accessions from Ethiopia. Botsw. J. Agric. Appl. Sci..

[11] Habtamu F.M., Wubeshet M., Mebrate D., Bekele S. (2021). Nutritional and phenolic profiles of leaves of fifteen Anchote (*Coccinia abyssinica*) accessions. Cogent Food Agric..

[12] Desta F.M. (2011). Phenotypic and Nutritional Characterization of Anchote [*Coccinia abyssinica* (Lam.) Cogn.] Accessions of Ethiopia. Master’s Thesis.

[13] Aga H., Badada K. (1997). Nutritional and antinutritional characteristics of anchote (*Coccinia abyssinica*). Ethiop. J. Health Dev..

[14] Adugna M.B., Yetenayet B.T., Tarekegn B.S., Sirawdink F.F. (2022). Anchote (*Coccinia abyssinica* [Lam.] Cogn.) powder, an underutilized indigenous crop, as a substitute to commercial pectin in the production of strawberry jam. Heliyon.

[15] Yohannes T.W. (2024). Anchote (*Coccinia abyssinica* (Lam.) Cogn.) Starch and Flour Modification Using Hydrothermal Treatments and Valorization as Functional Ingredients in the Development of Food Products. Ph.D. Dissertation.

[16] Melese A.D., Abera S., Mitiku D.H. (2021). Investigation of wheat—Anchote (*Coccinia abyssinica* (Lam.)) composite flours and baking temperature for cookies production. Food Res..

[17] Hailu R.G., Dassalegn D.J., Dinka M.B., Bezuayehu G.A., Kassaye T.S., Desta F.M. (2024). Nutritional and anti-nutritional composition of anchote (*Coccinia abyssinica*((Lam.) Cogn.) accessions in Ethiopia. J. Agric. Food Res..

[18] Tura S., Sandeep B.V., Sudhakar P., Aschalew T. (2018). Synthesis and characterization of zinc oxide nanoparticles using tuber extract of anchote (*Coccinia abyssinica* (Lam.) Cong.) for antimicrobial and antioxidant activity assessment. Open Nano.

[19] Desta F.M., Sentayehu A., Kibebew A., Mandefro N. (2024). Qualitative traits diversity in anchote [*Coccinia abyssinica* (lam.) cogn.] Accessions from Ethiopia. Asian J. Sci. Technol..

[20] Zerihun T., Meseret T.T., Bizuayehu T., Eleni S., Temesgen M.O. (2020). Genetic diversity in anchote (*coccinia abyssinica* (Lam.) Cogn.) using microsatellite markers. Curr. Plant Biol..

[21] Govindaraj M., Vetriventhan M., Srinivasan M. (2015). Importance of Genetic Diversity Assessment in Crop Plants and Its Recent Advances: An Overview of Its Analytical Perspectives. Genet. Res. Int..

[22] Mekonnen Y., Andargachew G., Bizuayehu T., Hewan D. (2024). Agro-morphological genetic diversity assessment of Amaranthus genotypes from Ethiopia based on qualitative traits. CABI Agric. Biosci..

[23] Pooran G. (2014). Breeding improvements in safflower (*Carthamus* tinctorius L.): A review. Aust. J. Crop Sci..

[24] Habte J., Kebebew A., Kassahun T., Kifle D., Zerihun T. (2020). Diversity in Qualitative and Quantitative Traits Reveals Huge Potential for the Improvement of an Orphan Crop Tef [*Eragrostis* tef (Zucc.) Trotter]. J. Exp. Agric. Int. (JEAI).

[25] Nigussie K., Solomon B., Gizachew H., Asnake F., Martin J. (2024). Agro-morphological characterization and comparative performance of Ethiopian and exotic lentil (*Lens culinaris* Medik) germplasm. Genet. Resour. Crop Evol..

[26] Guo L.J. (2013). Molecular Markers and Marker-Assisted Breeding in Plants. Plant Breeding from Laboratories to Fields.

[27] Sadia B., Awan F.S., Saleem F., Altaf J., Bin U.A., Nadeem M., Hameed S., Ashraf F., Nasir M., Maia R.T., Campos M.A. (2020). Exploring Plant Genetic Variations with Morphometric and Molecular Markers. Genetic Variation.

[28] Desta F.M., Sentayehu A., Kibebew A., Mandefro N. (2022). Quantitative Traits Diversity in Anchote (*Coccinia abyssinica* (Lam.) Cogn.)) Accessions from Ethiopia. Ethiop. J. Crop Sci..

[29] Sivasubramanian S.S., Madhava M.P. (1973). Genotypic and phenotypic variability in rice. Madras Agric. J..

[30] Robinson H.F., Comstock R.E., Harvey P.H. (1949). Estimates of heritability and the degree of dominance in corn. Agron. J..

[31] Zhi-Gang W., Wu J., Nitin M., Xiao-Qing B., Song-Lin C., Zheng-Ming T. (2015). Transcriptome analysis reveals flavonoid biosynthesis regulation and simple sequence repeats in yam (*Dioscorea alata* L.) tuber. BMC Genom..

[32] Hanna A., Chuntao S., Akwasi Y., Chunhua C., Shaoxia Y., Hongbo Z., Miao C. (2020). Flesh Color Diversity of Sweet Potato: An Overview of the Composition, Functions, Biosynthesis, and Gene Regulation of the Major Pigments. Phyton-Int. J. Exp. Bot..

[33] Badu M., Asho P., Patro T.S.K., Sasikala K. (2017). Studies on genetic variability, heritability, and genetic advance for growth, yield, and quality parameters among orange flesh sweet potato (*Ipomoea batatas* L. Lam.) genotypes. Int. J. Curr. Microbiol. Appl. Sci..

[34] Tewodros M., Firew M., Shimeles H., Endale G. (2023). Genetic Variability, Correlation and Path Analysis on the Storage Tuber Yield and Yield Components of Yam (*Dioscorea spp*.) from Southwest Ethiopia. Ethiop. J. Crop Sci..

[35] Oladosu Y., Rafii M.Y., Abdullah N., Malek M.A., Rahim H.A., Hussin G., Latif M.A., Kareem I. (2014). Genetic Variability and Selection Criteria in Rice Mutant Lines as Revealed by Quantitative Traits. Sci. World J..

[36] Prince E.N., Pangirayi B.T., Agyemang D., Eric Y.D., Paterne A.A., Afolabi A., Robert A., Asrat A. (2021). Genetic parameter estimation and selection in advanced breeding population of white Guinea yam. J. Crop Improv..

[37] Choudhary V.K., Kumar P.S., George J., Kanwat M., Saravanan R. (2011). Genetic Variability and Character Association in Taro (*Colocasia esculenta* (L.) Schott.) Under Mid-Hills of Arunachal Pradesh. J. Root Crops.

[38] Adhi S., Reddy R.V.S.K., Sujatha M., Pratap M. (2013). Genetic Variability Studies in F1 Generation of Tomato (*Solanum lycopersicon* L.). IOSR J. Agric. Vet. Sci..

[39] Lingaiah N., Satish C., Venkanna B., Rukmini D.K., Hari Y. (2020). Genetic Variability and Correlation Studies in Yield Traits of Elite Rice (*Oryza sativa* L.) Genotypes. Ind. J. Pure App. Biosci..

[40] Likeng-Li-Ngue B.C., Ibram A.A.M.M., Zenabou N., Zoa F.B., Fort M.N.L., Nathalie M., Seyum E.G., Bille H.N., Bell J.M. (2023). Genetic Variability, Heritability and Correlation of Some Morphological and Yield Components Traits in Potato (*Solanum tuberosum* L.) Collections. Am. J. Plant Sci..

[41] Rasheed A., Ilyas M., Khan T.N., Mahmood A., Riaz U., Chattha M.B., Al Kashgry N.A.T., Binothman N., Hassan M.U., Wu Z. (2023). Study of genetic variability, heritability, and genetic advance for yield-related traits in tomato (*Solanum lycopersicon* MILL.). Front. Genet..

[42] Alfredo A.C.A., Hillocks R.J., Thresh J.M., Bellotti A.C. (2002). Cassava Botany and Physiology. Cassava: Biology, Production and Utilization.

[43] Regessa M.D., Jiru N.C., Here A., Mulugeta N. (2023). Correlation and Mean Performance Evaluation of Sweet Potato (*Ipomoea batatas* (L.) Lam.) Genotypes Middle Awash Areas, Ethiopia. Adv. Crop Sci. Tech..

[44] Wubeshet T., Nesru T. (2021). Analysis on Production Potential of Anchote (*Coccinia abyssinica*) In East Wollega Zone, Oromia, Regional State, Ethiopia. Int. J. Adv. Res. Biol. Sci..

[45] Zeleke W., Hongxu D., Andrew H.P., Walelign W., Kassahun B. (2021). Genetic diversity, population structure, and selection signature in Ethiopian sorghum [*Sorghum bicolor* L. (Moench)] germplasm. G3 Genes Genomes Genet..

[46] Hair J.F., Anderson R.E., Tatham R.L., Black W.C. (1995). Multivariate Date Analysis with Readings.

[47] Aghaee M., Mohammadi R., Nabovati S. (2010). Agro-morphological characterization of durum wheat accessions using pattern analysis. Aust. J. Crop Sci..

[48] Rahman M., Munsur M. (2009). Genetic divergence analysis of lime. J. Bangladesh Agric. Univer..

[49] Basazinew D.G., Bizuayehu T.A., Wendawek A.M., Kebebew A.A. (2023). Morpho-Agronomic Characterization of Ethiopian Black cumin (*Nigella sativa* L.). Genotypes. Res. Sq..

[50] DZARC (2008). Annual Research Report, 2004/2005.

[51] ECPGR (2008). Minimum Descriptors for Cucurbit spp., Cucumber, Melon and Water Melon.

[52] International Plant Genetic Resources Institute (IPGRI) (2003). Descriptors for Melon (Cucumis melo L.).

[53] R Core Team (2024). R: A Language and Environment for Statistical Computing (Version 4.4.1).

[54] Oksanen J., Simpson G., Blanchet F., Kindt R., Legendre P., Minchin P., O’Hara R.B., Solymos P., Stevens M.H.H., Szoecs E. Vegan: Community Ecology Package. R Package Version 2, 2024; pp. 6–8. https://CRAN.R-project.org/package=vegan.

[55] Shannon C.E., Weaver W. (1949). The Mathematical Theory of Communication.

[56] Aravind J., Mukesh S.S., Wankhede D.P., Kaur V. (2023). augmentedRCBD: Analysis of Augmented Randomized Complete Block Designs. R Package Version 0.1.7. https://aravind-j.github.io/augmentedRCBD/.

[57] Wei T., Simko V.R. (2024). Package ‘Corrplot’: Visualization of a Correlation Matrix. (Version 0.95). https://github.com/taiyun/corrplot.

[58] Olivoto T., Lúcio A.D. (2020). metan: An R package for multi-environment trial analysis. Methods Ecol. Evol..

[59] Kassambara A., Mundt F. (2020). Factoextra: Extract and Visualize the Results of Multivariate Data Analyses. R Package Version 1.0.7. https://CRAN.R-project.org/package=factoextra.

[60] Venables W.N., Ripley B.D. (2002). Modern Applied Statistics with S.

[61] Wickham H. (2016). ggplot2: Elegant Graphics for Data Analysis.

[62] Maechler M., Rousseeuw P., Struyf A., Hubert M., Hornik K. (2023). cluster: Cluster Analysis Basics and Extensions. R Package Version 2.1.4. https://CRAN.R-project.org/package=cluster.

[63] Wickham H., François R., Henry L., Müller K. (2023). dplyr: A Grammar of Data Manipulation. R Package Version 1.1.4. https://CRAN.R-project.org/package=dplyr.

[64] Gower J.C. (1971). A general coefficient of similarity and some of its properties. Biometrics.

[65] Ward J.H. (1963). Hierarchical Grouping to Optimize an Objective Function. J. Am. Stat. Assoc..

[66] Maechler M. (2019). Finding groups in data: Cluster analysis extended rousseeuw Et. R Package Version.

[67] Van H., Hodgkin T., Brown A.H.D., Van Hintum T.J.L., Morales E.A.V. (1995). Hierarchical approaches to the analysis of genetic diversity in crop plants. Core Collection of Plant Genetic Resources.

[68] Raivo K. pheatmap: Pretty Heatmaps (Version 1.0.12). R Foundation for Statistical Computing 2020. https://cran.r-project.org/package=pheatmap.

[69] Mantel N. (1967). The detection of disease clustering and a generalized regression approach. Cancer Res..

[70] Galili T. (2015). dendextend: An R package for visualizing, adjusting, and comparing trees of hierarchical clustering. Bioinformatics.

