# Supplementary material for: Phenotypic Profiling of Anchote (Coccinia abyssinica (Lam.) Cogn.) Accessions Through Agro-Morphological and Physiological Markers
Source: Plants (Basel). 2025 Jul 28;14(15):2334. doi: 10.3390/plants14152334 (PMC12349358; doi:10.3390/plants14152334)
Supplement: Supplementary file 1 [file plants-14-02334-s001.zip › plants-3742776-supplementary.pdf]

Supplementary Materials

Table S1. list of Qualitative traits with their respective codes and description

Traits	code	scale	description
Root shape	RS		
Predominant root flesh color (widely observed color at first glance)	PRFC	1-9	1=white, 2=cream, 3=dark cream, 4=pale yellow, 5=dark yellow, 6=orange, 7=intermediate orange, 8=dark orange, 9=strongly pigmented
Secondary root flesh color (strips of anthocyanin within the predominant color)	SRFC	0-9	0=absent, 1=white, 2=cream, 3=dark cream, 4=pale yellow, 5=dark yellow, 6=pale yellow, 7=intermediate orange, 8=dark orange, 9=strongly pigmented
Vine color	VC	1-3	1=green, 2=light green, 3= purple
Leaf color	LC	1-3	1= green, 2= light green, 3= deep green
Ground coverage	GC	1-4	1= <50% LOW, 2= 50-70% medium 3= 70-90% high, 4= 90-100% very high

Table S2. List of quantitative agro- morphological and physiological traits recorded

Traits	code	Description
Petiole length(cm)	PL	Average Petiole length of 5 sample leaves per accessions were measured from base to tip of the petiole
Leaf length(cm)	LL	Average Leaf length of 5 sample leaves per accession were measured from the apex to the base of the leaf
Leaf diameter(cm)	LD	Average Leaf diameter of 5 sample leaves per accession measured from the widest point perpendicular to the longitudinal axis of the leaf
Internode length(cm)	INL	Average Inter node length of 5 sample vines per accession were obtained from maximum distance between two nodes
Vine length(m)	VL	Average length of vine of 5 sample vines per accession were measured from the base to the top of the main vine
Internode length to petiole length ratio(cm)	INL/PL	Ratio of internode length to petiole length
Fruit length (cm)	FL	Average maximum vertical length of the fruit of 5 sample fruits per accession

Fruit diameter (cm)	FD	Average longest distance of the fruit circle of 5 sample fruits per accession
Fruit length to diameter ratio (cm)	FL/FD	Ratio of fruit length to diameter
Number of seed per locule	NSPL	Number of seed from 5 sample locule per accession counted and averaged
Number of seed per fruit	NSPF	Seeds of 6 sample fruit per accession counted and averaged
Fruit weight (g)	FW	The weight of 5 sample fruit per accession weighed
Thousand seed weight(g)	TSW	Weight of one thousand seed per accession weighed
Seed yield (kg)	SY	Total Seed yield per accession weighed
Root number per plant	RNPP	Number of roots counted from 5 plant per accession and averaged
Root length(cm)	RL	The maximum vertical extent of the central axis of the roots of 5 root per accession were measured and averaged
Root diameter(cm)	RD	longest distance across the root circle of 5 root per accession were measured and averaged
Root length to diameter ratio (cm)	RL/RD	Ratio of root length to diameter calculated
Root weight per plot (Kg)	RWPP	Weight of roots per plot per accession were weighed
Root yield(t/ha)	RY	Total root yield per hectare converted based on average root weight per plant
Leaf area	LA	A product of leaf length and leaf diameter and K constant of 0.75
Leaf area index	LAI	measured by using CI-110 PLANT CANOPY IMAGER starting from 10:00 pm to 4:00 am of day time when there is sunlight.
Canopy density	CD	measured by using CI-110 PLANT CANOPY IMAGER starting from 10:00 pm to 4:00 am of day time when there is sunlight.
Gap fraction leaf area index	GFLAI	measured by using CI-110 PLANT CANOPY IMAGER starting from 10:00 pm to 4:00 am of day time when there is sunlight.
Chlorophyl content	ChC	fully expanded young leaves of three plants in each plot will be measured using a non-destructive, hand-held SPAD-502 chlorophyll meter
Normalized difference vegetative index	NDVI	measured using Trimble green shaker handheld crop sensor starts from 10:00 pm to 4:00 am of day time when there is sunlight.

Table S3. Passport data of the accessions collected

Acc	Zone	Woreda	Altitude	Acc	Zone	Woreda	Altitude
1	E.W	Sibu Sire	1834	81	E.W	Nunnu Kumba	2313
3	E.W	Wayu Tuka	1889	85	E.W	Leka Dullecha	2249
5	E.W	Wayu Tuka	1877	94	E.W	GudeyaBila	1949
6	E.W	Wayu Tuka	1825	95	E.W	GudeyaBila	1989
7	E.W	Wayu Tuka	1799	98	E.W	Gobbu Sayyo	1924
8	E.W	Wayu Tuka	1830	101	E.W	Gobbu Sayyo	1965
12	E.W	Chingi	1797	102	E.W	Gobbu Sayyo	1953

13	E.W	Chingi	1735	103	E.W	Gobbu Sayyo	1957
19	E.W	Leka Dullecha	1435	104	E.W	Gobbu Sayyo	1965
21	E.W	Digga	2178	105	W.W	Gimbi	1885
26	E.W	Leka Dullecha	2064	106	W.W	Gimbi	2006
27	E.W	Leka Dullecha	2170	109	W.W	Gimbi	2009
29	E.W	Leka Dullecha	2103	110	W.W	Gimbi	1919
31	E.W	Leka Dullecha	2078	111	W.W	Gimbi	1916
32	E.W	Leka Dullecha	2056	114	W.W	Gimbi	1995
35	E.W	Leka Dullecha	2244	115	W.W	Gimbi	1986
37	E.W	Leka Dullecha	2225	116	W.W	Gimbi	1860
38	E.W	Leka Dullecha	2240	117	W.W	Gimbi	1875
39	E.W	Leka Dullecha	2264	119	W.W	Gimbi	1917
41	E.W	Leka Dullecha	2227	120	W.W	Gimbi	2028
42	W.W	Gimbi	1776	122	W.W	Gimbi	1888
44	W.W	Gimbi	1650	123	W.W	Gimbi	1892
45	W.W	Gimbi	1857	124	W.W	Gimbi	1848
46	E.W	Digga	2129	126	W.W	Gimbi	1729
47	W.W	Gimbi	1843	127	W.W	Gimbi	1726
48	W.W	Gimbi	1867	129	W.W	Gimbi	1739
49	W.W	Gimbi	1870	130	W.W	Gimbi	1750
50	W.W	Gimbi	1888	131	W.W	Gimbi	1704
51	E.W	Digga	2199	132	W.W	Gimbi	1661
52	W.W	Gimbi	1820	134	W.W	Gimbi	1842
54	W.W	Gimbi	1837	139	W.W	Gimbi	2140
56	W.W	Gimbi	1864	140	W.W	Gimbi	2052
57	W.W	Gimbi	1888	141	W.W	Gimbi	1973
59	W.W	Gimbi	1943	142	W.W	Gimbi	1942
61	W.W	Lalo Asabi	1732	143	W.W	Gimbi	1889
64	W.W	Boji Dirmaji	1959	144	W.W	Gimbi	1891
68	W.W	Mana Sibu mandi	1583	145	W.W	Gimbi	1831
71	E.W	Leka Dullecha	2013	146	W.W	Gimbi	1806
72	E.W	Arjo	2427	147	W.W	Gimbi	1730
74	E.W	Leka Dullecha	1973	148	W.W	Gimbi	1793
76	E.W	Arjo	2430	149	W.W	Gimbi	1790
77	E.W	Leka Dullecha	2474	150	W.W	Gimbi	1824

Acc	Zone	Woreda	Altitude	Acc	Zone	Woreda	Altitude
151	W.W	Gimbi	1838	216	W.W	Mana Sibu Mandi	1558
152	W.W	Gimbi	1847	217	W.W	Mana Sibu Mandi	1688
153	W.W	Gimbi	1827	218	W.W	Boji Dirmaji	2004
154	W.W	Gimbi	1824	219	W.W	Boji Dirmaji	2000
157	W.W	Gimbi	1846	220	W.W	Boji Dirmaji	1965
160	W.W	Gimbi	1839	221	W.W	Boji Dirmaji	1944
162	W.W	Gimbi	1814	222	H.G.W	Horro	2399
164	W.W	Gimbi	1809	223	H.G.W	Horro	2392
165	W.W	Gimbi	1835	224	H.G.W	Horro	2381
166	W.W	Gimbi	1854	227	W.W	Boji Dirmaji	1964
167	W.W	Gimbi	1846	228	H.G.W	Horro	2371

168	W.W	Gimbi	1853	229	H.G.W	Horro	2369
170	W.W	Gimbi	1864	230	H.G.W	Guduru	2426
171	W.W	Gimbi	1832	234	E.W	Gute	1840
172	W.W	Gimbi	1875	236	Buno Bedelle	Bedelle	2030
173	W.W	Gimbi	1886	239	E.Gojjam	hulet eju	2441
174	W.W	Gimbi	1885	240	W.W	Gimbi	1885
175	W.W	Gimbi	1849	241	E.W	Sibu Sire	1815
176	W.W	Gimbi	1870	242	jimma	Dedo	2212
177	W.W	Gimbi	1845	244	Bench Madji	Andracha	1457
178	W.W	Gimbi	1841	245	H.G.W	Abay Chomen	2269
179	W.W	Gimbi	1831	246	Q.W	Dembi Dolo	1498
181	W.W	Gimbi	1830	247	Iluababor	Ale	1970
183	W.W	Gimbi	1766	248	E.W	Jimma Arjo	2297
184	W.W	Gimbi	1846	249	W.W	Gimbi	1630
187	W.W	Mana Sibu Mandi	1575	250	Q.W	Dembi Dolo	1541
188	W.W	Mana Sibu Mandi	1689	252	Iluababor	Ale	1952
189	W.W	Mana Sibu Mandi	1677	253	E.W	Guto wayu	2133
191	W.W	Mana Sibu Mandi	1688	254	E.W	Digga	2187
192	W.W	Mana Sibu Mandi	1629	255	W.W	Gimbi	1945
194	W.W	Mana Sibu Mandi	1555	258	E.W	Digga	2207
196	W.W	Mana Sibu Mandi	1611	259	W.Sh	Bako Tibbe	1658
199	W.W	Mana Sibu Mandi	1603	260	E.W	Digga	2174
200	W.W	Mana Sibu Mandi	1585	261	E.W	Jimma Arjo	2450
201	W.W	Mana Sibu Mandi	1622	262	E.W	Digga	2214
202	W.W	Mana Sibu Mandi	1590	264	E.W	Gudeya Bila	1877
203	W.W	Mana Sibu Mandi	1589	265	E.W	Gudeya Bila	1888
204	W.W	Mana Sibu Mandi	1574	266	E.W	Gudeya Bila	1886
206	W.W	Mana Sibu Mandi	1610	267	E.W	Gudeya Bila	1877
207	W.W	Mana Sibu Mandi	1567	268	E.W	Gudeya Bila	1877
208	W.W	Mana Sibu Mandi	1595	269	E.W	Gudeya Bila	1879
209	W.W	Mana Sibu Mandi	1610	271	E.W	Gudeya Bila	1883
210	W.W	Mana Sibu Mandi	1596	272	E.W	Gudeya Bila	1877
211	W.W	Mana Sibu Mandi	1622	273	E.W	Gudeya Bila	1923
212	W.W	Mana Sibu Mandi	1627	274	E.W	Gudeya Bila	1964
213	W.W	Mana Sibu Mandi	1652	275	H.G.W	Horro	3014
214	W.W	Mana Sibu Mandi	1543	276	H.G.W	Horro	2806
215	W.W	Mana Sibu Mandi	1536	277	H.G.W	Horro	2903

Acc	Zone	Woreda	Altitude	Acc	Zone	Woreda	Altitude
278	W.W	Mana Sibu Mandi	1590	346	Buno Bedelle	Makko	2078
280	W.W	Kiltu Kara	1817	348	Buno Bedelle	Makko	2087
281	W.W	Kiltu Kara	1793	349	Buno Bedelle	Makko	2079
282	W.W	Kiltu Kara	1747	351	Buno Bedelle	Makko	2101
283	W.W	Kiltu Kara	1694	352	Buno Bedelle	Makko	2266
284	W.W	Kiltu Kara	1717	354	Buno Bedelle	Chora	1944
285	W.W	Boji Dirmaji	1896	355	Iluababor	Hurumu	1803
287	W.W	Boji Dirmaji	1976	356	Iluababor	Hurumu	1822
289	W.W	Kiltu Kara	1839	358	Iluababor	Hurumu	1695
290	W.W	Boji Dirmaji	1853	359	Iluababor	Hurumu	1789
291	W.W	Boji Dirmaji	1805	363	Iluababor	Mettu	1699
293	W.W	Mana Sibu Mandi	1694	364	Iluababor	Mettu	1670

295	W.W	Mana Sibu Mandi	1718	365	Iluababor	Mettu	1710
296	W.W	Nejo	1913	366	Iluababor	Mettu	1668
298	W.W	Mana Sibu Mandi	1650	367	Iluababor	Mettu	1760
299	W.W	Mana Sibu Mandi	1661	368	Iluababor	Mettu	1686
300	W.W	Mana Sibu Mandi	1655	369	Iluababor	Mettu	1702
302	E.W	Limmu	2181	370	Iluababor	Mettu	1770
305	E.W	Limmu	2133	371	Iluababor	Mettu	1429
306	E.W	Limmu	2170	372	Buno Bedelle	Didessa	1659
307	E.W	Limmu	2151	373	Buno Bedelle	Didessa	1671
308	E.W	Limmu	2115	375	Buno Bedelle	Didessa	1432
310	E.W	Limmu	2122	376	Buno Bedelle	Didessa	1459
313	E.W	Gida Ayana	2060	379	Jimma	Gumay	2244
315	E.W	Gudeya Bila	2629	380	Buno Bedelle	Didessa	1470
316	E.W	Gida Ayana	2098	381	Buno Bedelle	Didessa	1427
317	E.W	Gudeya Bila	2194	382	Buno Bedelle	Didessa	1427
318	E.W	Gudeya Bila	2192	383	Buno Bedelle	Didessa	1450
319	E.W	Gudeya Bila	2315	384	H.G.W	Jimma Geneti	3016
320	E.W	Gudeya Bila	2339	385	H.G.W	Jimma Geneti	2945
321	E.W	Leka Dullecha	1892	386	H.G.W	Jimma Geneti	2929
322	E.W	Leka Dullecha	1887	391	E.W	Gudeya Bila	2042
323	E.W	Leka Dullecha	1863	393	E.W	Gudeya Bila	2063
324	E.W	Leka Dullecha	1847	395	E.W	Gudeya Bila	2049
325	E.W	Leka Dullecha	1899	396	E.W	Gudeya Bila	2251
326	E.W	Leka Dullecha	1871	397	E.W	Gudeya Bila	2289
327	E.W	Leka Dullecha	1825	399	E.W	Gudeya Bila	2329
328	E.W	Leka Dullecha	1855	401	E.W	Gudeya Bila	2192
329	E.W	Leka Dullecha	1854	402	E.W	Gudeya Bila	2252
330	E.W	Jimma Arjo	2478	404	E.W	Gudeya Bila	2332
333	E.W	Jimma Arjo	2313	405	E.W	Gudeya Bila	2338
334	E.W	Jimma Arjo	2424	406	E.W	Gudeya Bila	2216
336	E.W	Jimma Arjo	2294	408	E.W	Gudeya bila	2117
337	E.W	Jimma Arjo	2308	409	E.W	Gudeya bila	1974
338	E.W	Jimma Arjo	2456	410	E.W	Gudeya bila	2803
339	Buno Bedelle	Makko	2081	412	E.W	Gudeya bila	2257
340	Buno Bedelle	Makko	2061	414	H.G.W	Horro	2699
341	Buno Bedelle	Makko	2037	415	H.G.W	Horro	2676
343	Buno Bedelle	Makko	2072	416	H.G.W	Horro	3025
344	Buno Bedelle	Makko	2044	Check 1			
345	Buno Bedelle	Makko	2082	Check 2	Buno Bedelle	Didessa	1463
